# Neural heterogeneity controls computations in spiking neural networks

**DOI:** 10.1073/pnas.2311885121

**Published:** 2024-01-10

**Authors:** Richard Gast, Sara A. Solla, Ann Kennedy

**Affiliations:** ^a^Department of Neuroscience, Feinberg School of Medicine, Northwestern University, Chicago, IL 60611; ^b^Aligning Science Across Parkinson’s Collaborative Research Network, Chevy Chase, MD 20815

**Keywords:** heterogeneity, recurrent neural networks, neural dynamics, neural computation, mean-field models

## Abstract

The brain is composed of complex networks of interacting neurons that express considerable heterogeneity in their physiology and spiking characteristics. How does this neural heterogeneity influence macroscopic neural dynamics, and how might it contribute to neural computation? In this work, we use a mean-field model to investigate computation in heterogeneous neural networks, by studying how the heterogeneity of cell spiking thresholds affects three key computational functions of a neural population: the gating, encoding, and decoding of neural signals. Our results suggest that heterogeneity serves different computational functions in different cell types. In inhibitory interneurons, varying the degree of spike threshold heterogeneity allows them to gate the propagation of neural signals in a reciprocally coupled excitatory population. Whereas homogeneous interneurons impose synchronized dynamics that narrow the dynamic repertoire of the excitatory neurons, heterogeneous interneurons act as an inhibitory offset while preserving excitatory neuron function. Spike threshold heterogeneity also controls the entrainment properties of neural networks to periodic input, thus affecting the temporal gating of synaptic inputs. Among excitatory neurons, heterogeneity increases the dimensionality of neural dynamics, improving the network’s capacity to perform decoding tasks. Conversely, homogeneous networks suffer in their capacity for function generation, but excel at encoding signals via multistable dynamic regimes. Drawing from these findings, we propose intra-cell-type heterogeneity as a mechanism for sculpting the computational properties of local circuits of excitatory and inhibitory spiking neurons, permitting the same canonical microcircuit to be tuned for diverse computational tasks.

## Significance

Neurons are the basic information-encoding units in the brain. In contrast to information-encoding units in a computer, neurons are heterogeneous, i.e., they differ substantially in their electrophysiological properties. How does the brain make use of this heterogeneous substrate to carry out its function of processing information and generating adaptive behavior? We analyze a mathematical model of networks of heterogeneous spiking neurons and show that neural heterogeneity provides a previously unconsidered means of controlling computational properties of neural circuits. We furthermore uncover different capacities of inhibitory vs. excitatory heterogeneity to regulate the gating of signals vs. the encoding and decoding of information, respectively. Our results reveal how a mostly overlooked property of the brain—neural heterogeneity—allows for the emergence of computationally specialized networks.

The function of the nervous system is to process sensory information from the environment and generate adaptive behavioral responses that support survival. These computations require networks of neurons be able to reliably encode inputs, decode signals from the dynamics of other neural populations, and gate signals between sub-populations of neurons (1–3).

The neural populations that mediate these computations show a striking degree of inter-neuron heterogeneity, with cells varying in their structure, gene expression, and electrophysiological response properties (4–7). Scientists most often deal with cellular diversity by grouping neurons into dozens to hundreds of functionally or genetically defined “cell types,” and many studies have demonstrated that cell type diversity plays a key role in shaping neural computation (5, 8–11).

But there is clear experimental evidence that cell type boundaries are often fuzzy, with neurons of the same cell type showing considerable variance in their structure and response properties (12–15). In some instances, cells of a specific type have been demonstrated to have properties that vary along a continuum (16, 17). In addition to heterogeneity of physiological properties, neurons within a population can express diverse receptors for a particular neuromodulator, causing that neuromodulator to have heterogeneous effects on local neural activity (18, 19).

How can reliable neural computation be carried out given this variable substrate? It may be that biological computation is only sensitive to a small subset of physiological parameters (20), or that multiple distinct combinations of physiological properties give rise to similar network dynamics (21). However, it may also be that neural computation not only leverages distinct cell types, but also leverages within-type diversity of a neural population to shape that population’s dynamics and function.

In support of the hypothesis that within-population heterogeneity is important for neural computation, recent results suggest that the loss of within-type heterogeneity might underlie pathological cortical brain dynamics recorded in patients with epilepsy (22). This confirms previous results in mice where neural heterogeneity in the olfactory bulb was found to decrease neural synchronization and increase information transmission in the neural population (23). These results suggest that an important purpose of within-type heterogeneity is to make neural populations more resilient to pathological synchronization. Indeed, theoretical studies of heterogeneous neural network dynamics confirm that within-type heterogeneity can make networks less prone to synchronization (22, 24–27) (but see ref. 28 that reports that heterogeneity facilitates transitions to synchrony in a network model with purely excitatory neurons).

A reduction in pathological synchronization is not the only role that within-type heterogeneity can play. Computational studies of recurrent neural network models with within-type heterogeneity have found that heterogeneity can change the dimensionality of their intrinsic dynamics (27, 29), that it affects how neural populations respond to extrinsic inputs (30–32), and that it can cause phase transitions in the intrinsic network dynamics (33–35). Some of these findings suggest that the effect of within-type heterogeneity on population response properties is specific to neuron type, differing between excitatory and inhibitory neurons (12, 31, 32). It remains unclear how these effects on network dynamics relate to the functional role of heterogeneity in terms of the networks’ capacity to encode, transform, and gate signals in neural networks.

In this work, we develop a theoretical framework for studying the role of neural heterogeneity; the approach explicitly relates heterogeneity-induced changes in neural dynamics to changes in neural computation, in terms of the capacity of a network to encode, transform, and gate neural signals. To this end, we derive mean-field equations for spiking neural networks (SNNs) that express heterogeneity in their spike thresholds. We systematically examine the effects of spike threshold heterogeneity on the network dynamics and computations, using methods from dynamical systems theory and machine learning. Our results suggest that spike threshold heterogeneity does not only serve to desynchronize neural circuit dynamics; it also serves to support distinct computational functions in inhibitory and excitatory neurons. Whether more or less heterogeneity is beneficial for the computational abilities of neural circuits depends on the computational demands that the circuit faces.

## Results

We incorporate heterogeneity in a spiking neural network model by introducing quenched disorder in model parameters. Quenched disorder on a model parameter implies that the values taken by the parameter are sampled from a chosen probability distribution and randomly assigned to each individual neuron in the network. Once assigned, the parameter value assigned to each neuron remains fixed, constant over time.

To study the effects of within-type heterogeneity on a neural population, we must first identify relevant model parameters that capture aspects of within-type heterogeneity in biological neurons. Previous work on SNNs have considered the input current I as a quenched, distributed variable (26, 35–37). Since the actual value of input currents is hard to measure experimentally, the underlying distribution of this random variable cannot be empirically characterized; this difficulty interferes with the ability to compare the predictions of a neural network model to the actual dynamics of biological neural networks. Moreover, the distribution of I values is not an intrinsic property of the network, as it reflects a property of external input sources.

Electrophysiological properties of individual model neurons, such as their membrane capacitance, membrane resistance, resting potential, or spiking threshold, capture variations in the structural composition of the cell membrane across neurons of the same type (38) as well as across distinct neural types. Differences in the electrophysiological properties of neurons result in differences in their spiking responses to a given synaptic input. We thus identify heterogeneity in the electrophysiological properties of model neurons as an appropriate scenario for studying the effect of genetic or anatomical sources of within-type heterogeneity in biological networks.

Specifically, we consider the spike threshold of individual neurons as a quenched, distributed parameter. Spike thresholds are known to capture properties of the ion channel distribution in the cell membrane that determine the sensitivity of the cell to synaptic inputs (39). Thus, the heterogeneity of spike thresholds has often been linked to the typical sigmoidal response curve of neural populations (22, 40, 41). Importantly, spike thresholds can be directly determined from single cell recordings; their inclusion in our model thus allows for a direct comparison of the effects of within-type heterogeneity between neural network models and biological networks.

To determine biologically reasonable ranges of spike threshold heterogeneity, we referenced the NeuroElectro database (42). Reported spike thresholds vary between −65 and −10 mV, with standard deviations of spike thresholds of a single cell type varying from ≈1 mV (43) to more than 10 mV (44). These data reveal substantial differences in spike threshold heterogeneity across cell types and brain regions. For example, cerebellar Purkinje cells have been reported to express much more homogeneous spike thresholds than CA1 pyramidal cells (45), whereas recordings from cortical Martinotti interneurons in rodents have revealed SDs of spike thresholds that varied between 2 and 8 mV across cortical layers (46).

Below, we study how spike threshold variations within this range affect the dynamics and function of neural populations.

### Mean-Field Equations for Networks of Heterogeneous Spiking Neurons.

To study the effect of within-type heterogeneity on populations of one or more functional cell types, we model each population of a specific cell type as a network of coupled Izhikevich (IK) neurons (4) with parameters tuned to replicate the spiking behavior of the specific cell type. The network model takes the form

[1] $$C{\dot{v}}_{i}=k(v_{i}-v_{r})(v_{i}-v_{\theta ,i})-u+I(t)+gs(E-v_{i})$$

[2] $$\tau_{u}\dot{u}=-u+b(-v_{r}+\frac{1}{N}\sum_{j=1}^{N}v_{j})+\tau_{u}\kappa r,$$

[3] $$\tau_{s}\dot{s}=-s+J\tau_{s}r,$$

[4] $$r(t)=\frac{1}{N}\sum_{j=1}^{N}\underset{k\backslash t_{j}^{k}\leq t}{\sum }\delta (t-t_{j}^{k}).$$

Here, $v_{i}$ represents the membrane potential of the ith neuron in the network; s reflects the post-synaptic activation of neurons in the network, subject to the low-pass filtering effects of synaptic integration; u is a global recovery variable; and r(t) is the firing activity, averaged across all neurons in the network. We provide additional details on these equations and their parameters in *Materials and Methods*, Section A.

To introduce within-population spike threshold heterogeneity, we assume that the spike thresholds $\left\{v_{\theta ,i}\right\}$ are distributed according to a Lorentzian probability distribution parameterized by a center at ${\overset{¯}{v}}_{\theta }$ and half-width-at-half-maximum $\Delta_{v}$, as described by the density function

[5] $$\begin{array}{c} p\left(v_{\theta }\right)=\frac{1}{\pi }\frac{\Delta_{v}}{{\left[v_{\theta }-{\overset{¯}{v}}_{\theta }\right]}^{2}+\Delta_{v}^{2}}. \end{array}$$

An increase of $\Delta_{v}$ yields a higher variance of spike thresholds across neurons is in the network; the width $\Delta_{v}$ of the Lorentzian probability density thus determines the extent of within-type heterogeneity. Under the Lorentzian assumption, the following set of mean-field equations can be derived analytically:

[6] $$C\dot{r}=\frac{{\text{Δ}}_{v}k^{2}\sigma_{v}}{\pi C}(v-v_{r})+r[k(2v-v_{r}-{\bar{v}}_{\theta })-gs],$$

[7] $$\begin{array}{l} C\dot{v}=kv(v-v_{r}-{\bar{v}}_{\theta })-\pi Cr(\Delta_{v}\sigma_{v}+\frac{\pi C}{k}r)\\ \;\;\;\;\;\;\;\;\;\;\;\;\;+kv_{r}{\bar{v}}_{\theta }-u+I+gs(E-v), \end{array}$$

[8] $$\tau_{u}\dot{u}=b(v-v_{r})-u+\tau_{u}\kappa r,$$

[9] $$\tau_{s}\dot{s}=-s+\tau_{s}Jr,$$

where $\sigma_{v}\equiv \;\text{sign}\left(v-v_{r}\right)$. A more detailed derivation of these mean-field equations can be found in *SI Appendix*.

The four differential equations Eqs. **6**–**9** capture the macroscopic dynamics of the Izhikevich neuron network of Eqs. **1**–**3**. We can examine how within-type heterogeneity affects the dynamics of SNNs by systematically studying the effects of $\Delta_{v}$ on the dynamics of the mean-field equations.

An examination of Eqs. **6**–**7** indicates that $\Delta_{v}$ shapes the dynamics of both r and v; this effect is multiplicative and hence depends on the value of these two state variables. The effect of threshold heterogeneity in our model thus depends on the dynamic regime of the population. When the population membrane potential v is close to its resting value $v_{r}$, $\Delta_{v}$ barely affects the population firing activity, since $v-v_{r}$ in Eq. **6** vanishes and r in Eq. **7** is small. However, when the population is in a depolarized or hyperpolarized state, the effect of $\Delta_{v}$ is harder to predict, since it affects Eqs. **6** and **7** in different ways. The effects of neural heterogeneity in our mean-field model thus differ qualitatively from those found in previous work (36, 47, 48), where neural heterogeneity was implemented as variance over I and only led to a shift in the average firing activity of the population.

### Neural Heterogeneity in Populations of Neurons Composed of a Single Cell Type.

To improve our understanding of within-type heterogeneity on the dynamics of neural populations, we studied the effects of changes in the range of spike threshold heterogeneity quantified by $\Delta_{v}$ on the mean-field dynamics of SNNs. To validate our mean-field model, we contrast our findings with numerical bifurcation analysis of simulations of SNNs, details of which are provided in *Materials and Methods*, Section B. Specifically, we compare our mean-field predictions to simulations of SNNs with either Lorentzian or Gaussian spike-threshold distributions and with sparse synaptic connectivity (coupling probability of 20%), to determine the extent to which the mean-field assumptions of all-to-all connectivity and Lorentzian-distributed spike thresholds matter for prediction of network dynamics. We will first examine predictions of our mean-field model and then discuss the model’s alignment with simulation results from SNNs.

**Fig. 1.**
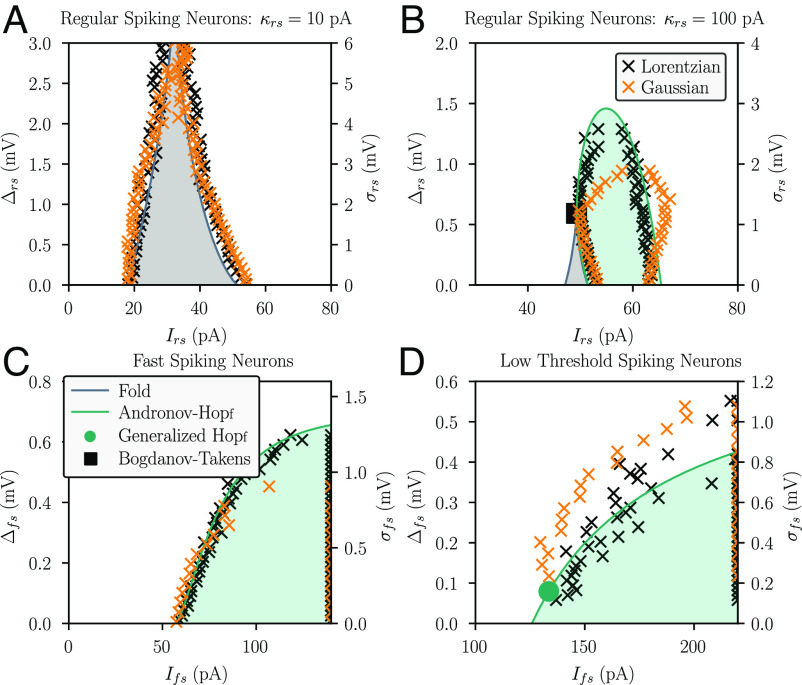
Heterogeneity linearizes neural population dynamics. (*A*–*D*) Two-dimensional (2D) bifurcation diagrams are depicted for different cell types. Regions colored in gray and green depict bistable and oscillatory regimes, respectively. The black and orange crosses depict approximate bifurcation points, estimated from the dynamics of simulated SNNs with a Lorentzian and a Gaussian distribution of the spiking thresholds, respectively. Spiking neural network dynamics were obtained from simulating networks of N = 1,000 neurons connected by sparse, random couplings (coupling probability of 20%) The y-axis on the *Left* (*Right*) depicts the width of the Lorentzian (Gaussian) distribution used to generate the SNNs that result on the bifurcation points shown by the black (orange) crosses. (*A*) Excitatory regular-spiking neurons with low spike-frequency adaptation ($\kappa_{\mathrm{rs}}=10$ pA). (*B*) Excitatory regular-spiking neurons with high spike-frequency adaptation ($\kappa_{\mathrm{rs}}=100$ pA). (*C*) Inhibitory fast-spiking neurons. (*D*) Inhibitory low-threshold-spiking neurons.

First, we examined the effects of spike threshold heterogeneity on the dynamics of a single population of excitatory, regular-spiking neurons. Such a population can either be in a monostable asynchronous regime, a bistable asynchronous regime, or a regime of synchronous oscillations (Fig. 1 *A* and *B*).

**Fig. 2.**
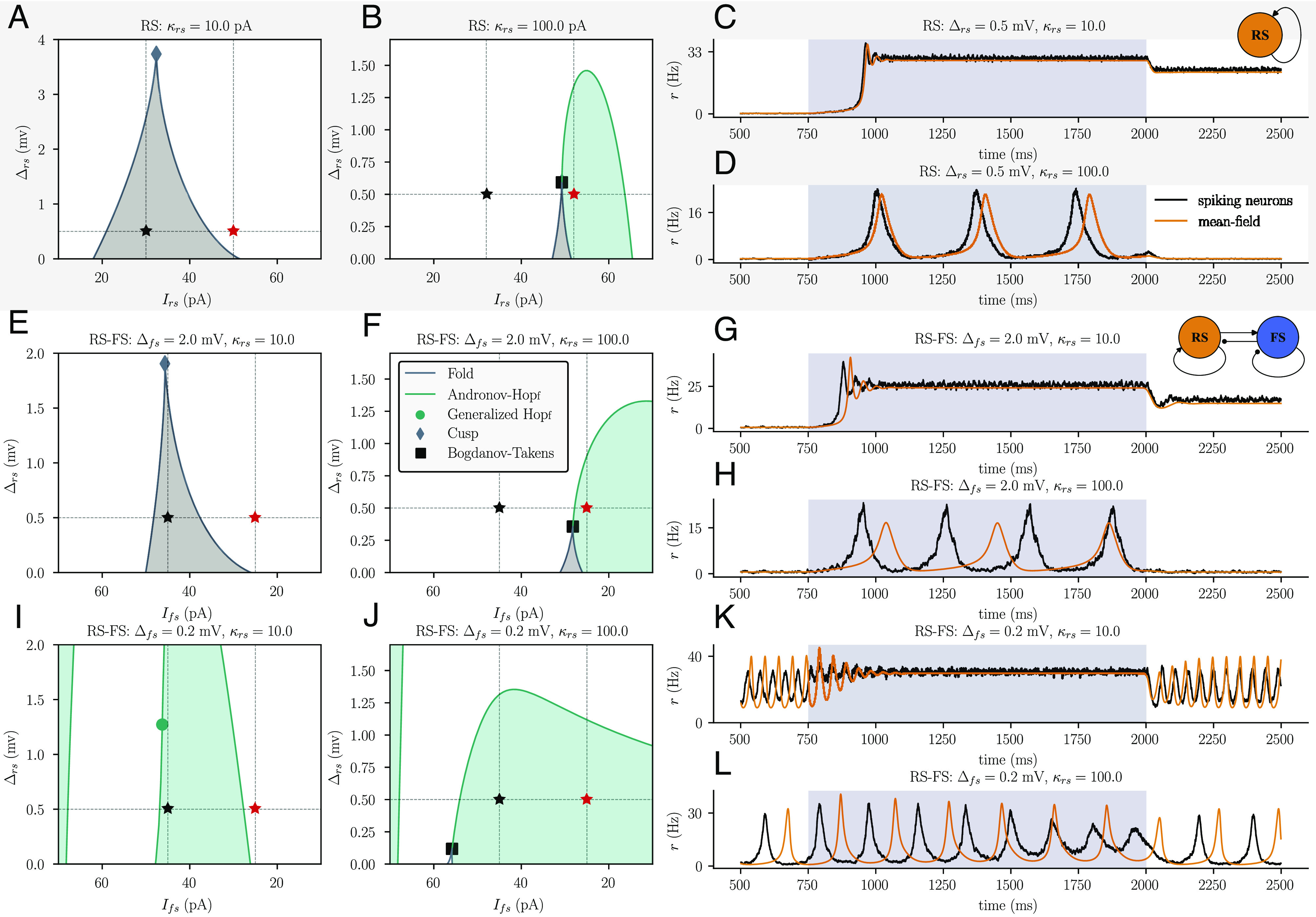
Homogeneous inhibitory interneurons overwrite the bifurcation structure of excitatory neurons. 2D bifurcation diagrams: Regions colored in gray and green depict bistable and oscillatory regimes, respectively. The black and red stars indicate the value of the input current used during low input (white, t<750 ms and t>2,000 ms) and high input (gray-blue, 750 ms <t<2,000 ms) regimes, respectively. The effect of low and high inputs in the firing dynamics is shown in the third column. (*A* and *B*) 2D bifurcation diagrams for a single population of regular-spiking neurons (RS) with weak ($\kappa_{\mathrm{rs}}=10$ pA) and strong ($\kappa_{\mathrm{rs}}=100$ pA) spike frequency adaptation, respectively. (*C* and *D*) Firing dynamics of the RS population with weak (*C*) vs strong (*D*) spike frequency adaptation. Spiking dynamics were obtained from a network with N = 2,000 neurons with sparse, random coupling (coupling probability of 20%). (*E* and *F*) 2D bifurcation diagrams for a two-population network of regular-spiking and fast-spiking (FS) neurons with high FS neuron heterogeneity. The bifurcation diagrams resemble those of the one-population model (compare to *A* and *B*). Note that the direction of the x-axis is flipped in comparison to *A* and *B*, to account for the fact that increases in $I_{\mathrm{rs}}$ excite the RS population, whereas increases in $I_{\mathrm{fs}}$ cause inhibition of the RS population due to the inhibitory nature of the FS-to-RS projection. The input to the RS population was fixed at $I_{\mathrm{rs}}=60$ pA. (*G* and *H*) RS firing dynamics in the two-population model with high FS neuron heterogeneity, which closely resembles that of the single population model (compare to *C* and *D*). Spiking dynamics were obtained from simulations of a network of N = 2,000 RS neurons and N = 2,000 FS neurons with sparse, random coupling (coupling probability of 20%). (*I* and *J*) Same as *E* and *F* but for low FS neuron heterogeneity. (*K* and *L*) Same as *G* and *H* but for low FS neuron heterogeneity.

In the monostable asynchronous regime, only a single stable state exists, and the dynamical regime of the network always converges to it after a sufficiently long time (mathematically, as t→∞). The bistable asynchronous regime is characterized by the co-existence of two stable states: a stable node that represents a quiescent state and a stable focus that represents a persistent spiking state. The initial state of the network determines whether it converges to the quiescent or the persistent spiking state, and inputs to the system can shift the network state from one stable point to the other. The domains of attraction of these two states depend on the location of an unstable saddle point that separates the stable node from the stable focus (see also Fig. 2*C* where we show a transition of the network between those two states). Finally, the synchronous oscillation regime arises from the interaction of fast, recurrent excitation with spike frequency adaptation (48).

The parameter κ in Eq. **2** controls the level of spike frequency adaptation, and thus the propensity of the system to enter the synchronous oscillation regime. To cover the space of possible network regimes, we therefore examined the network dynamics for two different levels of $\kappa_{\mathrm{rs}}$: one that allows for oscillations to exist ($\kappa_{\mathrm{rs}}=100$ pA) and one that does not ($\kappa_{\mathrm{rs}}=10$ pA).

We find that changing the spike threshold heterogeneity $\Delta_{\mathrm{rs}}$ of the excitatory neurons affects the transition between these regimes, a transition that can be induced by changes in the extrinsic input $I_{\mathrm{rs}}$ (Fig. 1 *A* and *B*). The width of the bistable asynchronous regime (given by the horizontal distance between the fold bifurcation curves in Fig. 2*A*) decreases as the spike threshold heterogeneity increases, until the bistable regime vanishes in a cusp bifurcation. Similarly, the width of the synchronous regime (given by the horizontal distance between the Hopf bifurcation curves in Fig. 2*B*) decreases as the spike threshold heterogeneity increases, until the synchronous regime vanishes in a supercritical Hopf bifurcation.

Next, we studied the effects of spike threshold heterogeneity on populations of inhibitory neurons, which are thought to play a central role in maintaining a balanced and yet responsive dynamical regime in the cortex (49). To account for the different time scales of inhibition reported for cortical interneurons (50), we analyzed fast spiking as well as low-threshold-spiking neurons, two interneuron types with distinct membrane time constants (39). Both types of interneurons can either be in an asynchronous or a synchronized, oscillatory regime; the transitions between these states occur via an Andronov–Hopf bifurcation (Fig. 1 *C* and *D*). As spike threshold heterogeneity is increased, the oscillatory regime becomes narrower until it eventually vanishes.

The disappearance of bistable and synchronized oscillatory states indicates that spike threshold heterogeneity linearizes the dynamics of neural populations composed of a single cell type, independent of whether that cell type is excitatory or inhibitory.

We find that the results that follow from the analysis of the mean-field equations agree with the dynamics of simulated SNNs with similar parameters (Fig. 1*A*–*D*). To perform SNN simulations with either Lorentzian or Gaussian distributions of spike thresholds, we estimated the location of fold and Andronov–Hopf bifurcations in the SNN dynamics by considering the response of the networks to a slowly ramping input (see the *Materials and Methods*, Section B for details). We used both Gaussian and Lorentzian heterogeneity distributions to test whether our results depend on the particularly long tails of the Lorentzian distribution. Fig. 1 shows the bifurcation structure of SNNs with either spike threshold distribution. We find that the bifurcation structure of the SNNs is qualitatively well captured by the bifurcation diagrams obtained from the mean-field equations (also see *SI Appendix*, Figs. S2–S4 for spiking dynamics and firing rate distributions of SNNs with Gaussian and Lorentzian spike threshold distributions).

These results illustrate the robustness of the mean-field approximation, as the SNN simulations were carried out in networks with sparse random connectivity as opposed to the all-to-all connectivity assumed in the derivation of the mean-field equations (see the *Materials and Methods*, Section A for details).

### Neural Heterogeneity in Excitatory-Inhibitory Networks.

In much of the brain, inhibitory neurons are local interneurons that act on neighboring excitatory neurons to modify their dynamics (5, 8). We asked how spike threshold heterogeneity in local inhibitory interneurons might alter the responses of recurrently coupled excitatory projection neurons. To address this question, we extend our model Eqs. **1**–**3** to a two-population model of coupled excitatory regular-spiking and inhibitory fast-spiking neurons (see the *Materials and Methods*, Section A for details). We analyzed how the fast-spiking interneurons modulate the bifurcation structure of the regular-spiking neuron population and how this modulation depends on the degree of heterogeneity of the fast-spiking population. We applied bifurcation analysis to the two-population model for different levels of $\Delta_{\mathrm{fs}}$ and compared the resulting bifurcation diagrams to the bifurcation structure of the isolated regular-spiking excitatory population. To again account for the full range of dynamics expressed by the regular-spiking population, we repeated this procedure for low and high spike-frequency adaptation (see Fig. 1 *A* and *B* for $\kappa_{\mathrm{rs}}=10$ pA and $\kappa_{\mathrm{rs}}=100$ pA, respectively).

When the spike threshold heterogeneity of the inhibitory population is low, the bistable asynchronous regime observed in the one-population model of purely excitatory neurons ceases to exist, and synchronous oscillations become the dominant dynamic regime (Fig. 2 *I* and *J*). However, when the spike threshold heterogeneity of the inhibitory population is increased, the behavior of the two-population model begins to revert to that of the one-population model (Fig. 2 *E* and *F*). Specifically, the input $I_{\mathrm{fs}}$ to inhibitory interneurons induces the same phase transitions in excitatory activity that we found in the excitatory-only model. Changes in the spike threshold heterogeneity of the excitatory population produce qualitatively similar changes in their dynamic regime (compare Fig. 2 *E* and *F* to Fig. 2 *A* and *B*).

We confirmed the predictions obtained from the mean-field equations via simulations of SNNs with sparse, random coupling (coupling probability of 20%). We chose to simulate networks with sparse coupling rather than all-to-all coupling to demonstrate that our mean-field results generalize to biologically more plausible network architectures (see the *Materials and Methods*, Section A for details). The average firing rate dynamics obtained from these SNNs closely match the firing rate dynamics predicted by the mean-field equations (Fig. 2).

To determine whether the observed effect of inhibitory heterogeneity is specific to fast-spiking interneurons, we added to the model an inhibitory interneuron population with substantially different electrophysiological properties: low-threshold-spiking interneurons (5, 39). As above, we found that homogeneous low-threshold-spiking interneurons drove synchronized oscillations that masked the bifurcation structure of the excitatory population and that increasing the spike threshold heterogeneity of these interneurons recovered that structure (Fig. 2 and *SI Appendix*, Fig. S5).

Note that the widths of the spike threshold distributions chosen for each of the three subpopulations match spike threshold variances reported in the literature; this choice emphasizes the relevance of our findings to understanding the dynamic organization of biological neural networks. Specifically, the values of $\Delta_{\mathrm{fs}}$ and $\Delta_{\mathrm{lts}}$ used for *SI Appendix*, Fig. S5 were fitted to resemble sample variances reported for recordings from cortical fast-spiking and low-threshold-spiking neurons in rodents (43, 46); the fitting method is described in *SI Appendix* (see *SI Appendix*, Fig. S1 for the obtained fits).

We conclude that heterogeneous inhibitory interneurons preserve the bifurcation structure of the excitatory population, whereas homogeneous inhibitory interneurons overwrite this bifurcation structure and move the system toward highly synchronized dynamics. Within-type heterogeneity of inhibitory interneurons is therefore an important control variable for their role in computation by neural circuits. Previous attractor-based theories of brain function have posed inhibitory synchronization as a means of gating signal propagation in mesoscopic brain circuits (11). Here, we extend this theory of inhibitory neurons’ role in neural computation to show how inhibitory gating could be tuned by increasing or decreasing the spike threshold heterogeneity of the inhibitory population. Such tuning of spike threshold heterogeneity could be achieved either developmentally or by heterogeneous action of neuromodulatory input (18, 19).

Attractor-based theories of neural computation also suggest that excitatory projection neurons are the central elements that implement signal encoding and transformation functions in extended brain networks. Does within-type heterogeneity play a similarly important role for encoding and transformation functions as it appears to play for gating functions? In the next sections, we address this question by examining the influence of spike threshold heterogeneity on the signal encoding and transformation capacities of recurrently coupled excitatory neurons.

### Neural Heterogeneity Affects Encoding Capacities of Multistable Networks.

Our mean-field analysis shows that within-type heterogeneity decreases the extent of the bistable regime of excitatory populations and linearizes their responses, even in networks of multiple cell types. We hypothesized that this mechanism could have important repercussions for models of neural computation that rely on the nonlinearity of population dynamics. For example, network-level multistability is a crucial component of many theoretical models of information encoding via persistent neural activity (11, 51, 52). Can networks of excitatory neurons still function as multistable information encoding circuits in the face of heterogeneity?

**Fig. 3.**
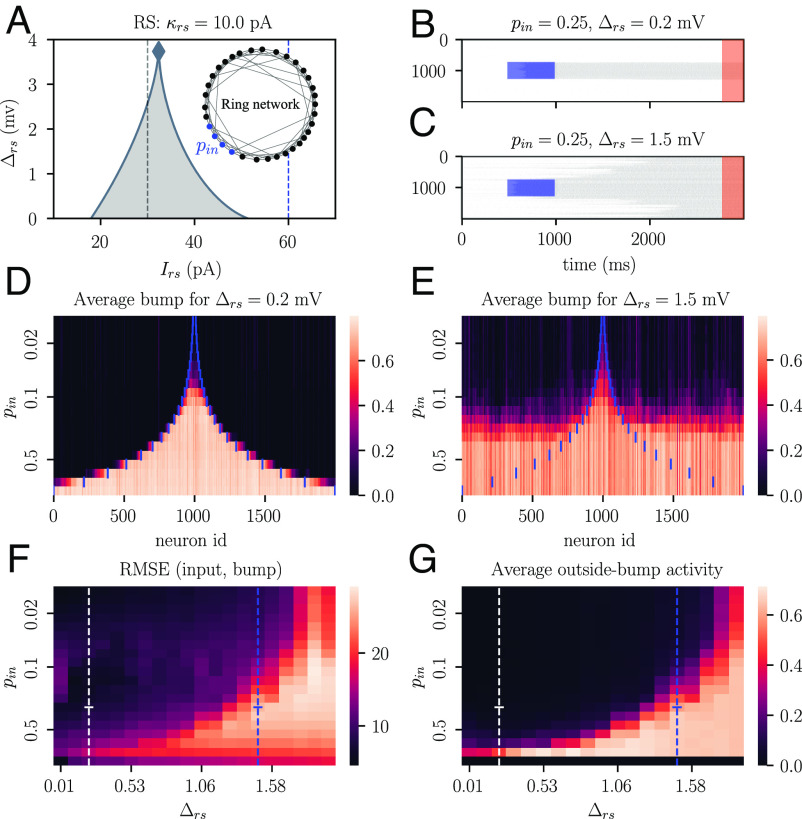
Spike threshold heterogeneity impairs the retention of spatially localized activity in a ring network. (*A*) 2D bifurcation diagram of the regular-spiking neuron population (same as in Fig. 2*A*). Vertical lines indicate the input drive to the network at baseline (gray, $I_{\mathrm{rs}}=30$ pA) and during extrinsic stimulation (blue, $I_{\mathrm{rs}}=60$ pA). The inset shows the structure of the ring network; the stimulated section of the ring is shown in blue. (*B* and *C*) Spiking activity as a function of time for an homogeneous and an heterogeneous network, respectively; the simulated networks consist of N = 2,000 neurons with sparse coupling (20%). The blue-shaded regions show the application of a rectangular pulse of extrinsic stimulation to a ring segment whose width is $p_{\mathrm{in}}=0.25$ of the length of the ring. The orange-shaded region depicts the asymptotic time interval used to compute the average neural activity shown in (*D*–*G*). (*D* and *E*) Persistent activity of each neuron along the ring during the orange-shaded test interval depicted in (*B* and *C*). Results are shown as a function of the width $p_{\mathrm{in}}$ of the input stimulation. The neural activity is normalized to the maximum observed spike rate and averaged over 10 random initializations of the network. The blue vertical lines in each row delimit the section of the network that received extrinsic input. (*F*) Root mean squared error between the activity of the network during the application of the extrinsic input and the activity of the network during the test interval, as a function of both input width $p_{\mathrm{in}}$ and degree of population heterogeneity $\Delta_{\mathrm{rs}}$. (*G*) Normalized spiking activity during the test interval, averaged over the neurons that did not receive extrinsic stimulation.

To address this question, we again focused on the characterization of population activity in spiking neural network models. First, we studied the effect of within-type heterogeneity on a network’s capacity to encode an input signal within a region of spatially localized persistent activity (referred to here as an “activity bump”). To impose a notion of proximity and spatial localization in the network model, we considered a ring network of sparsely coupled regular-spiking neurons (Fig. 3*A*) whose parameters were chosen to initialize the network in its bistable regime. In this network, stimulating one section of the ring induces elevated neural firing that persists after stimulation offset. The location of persistent firing within the ring can be used to encode a working memory trace of the stimulation provided it maintains stimulus specificity.

To evaluate the heterogeneous ring network’s capacity to encode its input history, we applied extrinsic stimulation to a section of the ring and studied the network’s capacity to retain the location and width of the stimulated section (see the corresponding *Materials and Methods*, Section C for more details). We repeated this analysis while systematically varying the width of the stimulated section as well as the level of spike threshold heterogeneity within the population. As expected, a homogeneous network ($\Delta_{\mathrm{rs}}=0.2$ mV) can stably encode stimulus identity via a stimulus-specific bump of persistent activity for a wide range of stimulus widths (Fig. 3 *B* and *D*). In contrast, a more heterogeneous network ($\Delta_{\mathrm{rs}}=1.5$ mV) is capable of persistent firing after stimulation but fails to maintain information about the stimulus location for all but a small range of narrow stimulus widths (Fig. 3 *C* and *E*). The persistent state is increasingly prone to diffusing through the network as spike threshold heterogeneity is increased (Fig. 3 *F* and *G*).

These results demonstrate a relationship between the bifurcation structure of a network and its capacity to maintain spatially localized activity required for stimulus encoding. At the mean-field level, increased spike threshold heterogeneity in the network reduces the width of its bistable regime (Fig. 3*A*). Because of this reduced bistability range, small changes in extrinsic input can push the system over a fold bifurcation, moving it from a low to a high activity regime or vice versa. In addition, heterogeneous networks operating within a narrow bistable regime are more vulnerable to input noise, since noisy inputs are more likely to induce phase transitions that move the network out of its bistable regime. We conclude that heterogeneity results in a loss of spatially localized dynamics and reduced spatial encoding capacities in the ring network. In brain structures that require spatial localization of neural activity for stimulus encoding, we would therefore predict a tendency toward reduced neural heterogeneity, or the introduction of compensating mechanisms such as sparse, structured connectivity to maintain stimulus identity.

### Neural Heterogeneity Increases the Computational Capacity of Neural Populations.

**Fig. 4.**
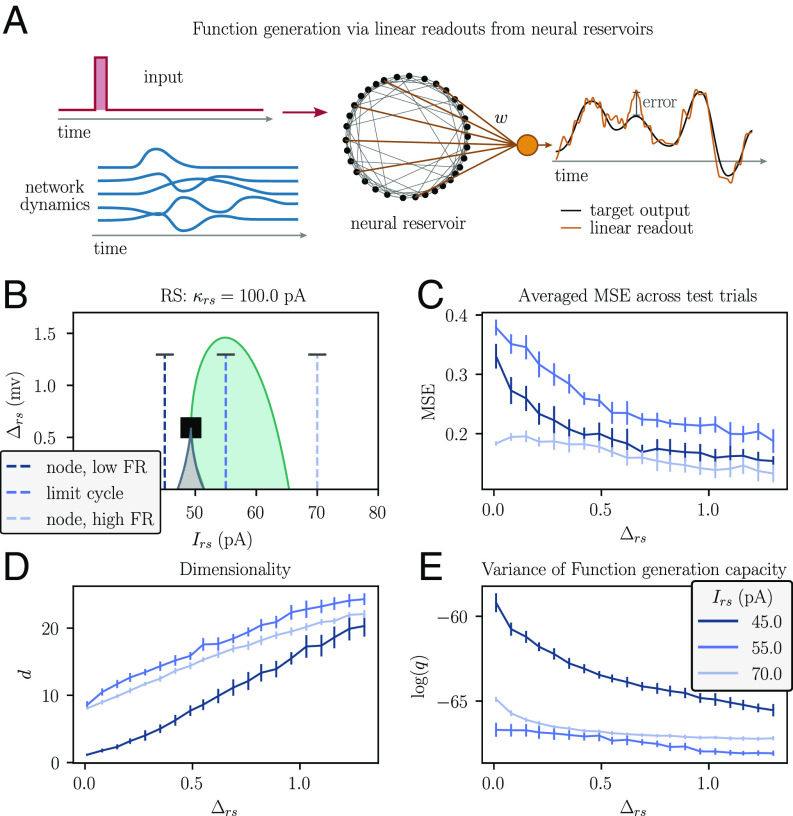
Spike threshold heterogeneity affects the function generation properties of spiking neural networks. (*A*) Reservoir computing architecture used for function generation. A pulse is fed into a recurrent neural network and a linear readout is trained to minimize the mean squared error between a target time-dependent function and a network output obtained as a linear combination of the stimulus-evoked neural dynamics within the network. (*B*) 2D bifurcation diagram of the regular-spiking neuron population (same as in Fig. 2*B*). The vertical dashed lines mark the three different dynamical regimes for which the results in (*C*–*E*) are depicted: $I_{\mathrm{rs}}=45$ pA, $I_{\mathrm{rs}}=55$ pA, and $I_{\mathrm{rs}}=70$ pA. The height of these lines indicates the range of values of $\Delta_{\mathrm{rs}}$ for which the results in (*C*–*E*) were obtained. (*C*) Mean squared error between the network readout signal and the target signal, averaged over time and across all trials of the test dataset. (*D*) Dimensionality d of the network dynamics, calculated from spike train correlations between neurons in the network. (*E*) Normalized variance of the network response kernel averaged over trials indicates the reliability of the function generation capacities of the network. See the *Materials and Methods*, Section D for a detailed explanation of how the quantities depicted in (*C*–*E*) were calculated. Vertical lines shown in (*C*–*E*) depict the SD across trials.

The previous section suggests that heterogeneity interferes with encoding functions that rely on population-level multistability. The transformation of information is a second core function that neural networks may support via their collective dynamics (53). The coordinated activity of recurrently coupled neurons can enable neural populations to extract important features of their input and produce temporally extended outputs that are transformations of those features. In this section, we study how within-type heterogeneity affects the signal transformation capacity of an excitatory neural network by analyzing its ability to reliably compute input–output transformations via its dynamics (Fig. 4*A*).

We initialized a ring network of sparsely coupled regular-spiking neurons in three distinct dynamic regimes: i) an asynchronous, quiescent regime, ii) a synchronous, oscillatory regime, and iii) an asynchronous, persistently active regime (Fig. 4*B*). We then applied a short pulse of extrinsic stimulation to a small section of the ring in each of these three different states of the intrinsic population dynamics and recorded the network response to the stimulation. We tested the capacity of the network to generate an arbitrary time-dependent target output in the firing rate of an output unit. The output unit used learned weights to read the activity of the neurons within the network, while synaptic weights between network neurons were kept fixed. This approach is based on the concept of reservoir computing (54–56); a detailed description of the reservoir computing framework is provided in the corresponding subsection within *Materials and Methods*, Section D.

The capacity of the network to reliably generate a desired time-dependent output increased with neural heterogeneity in all three dynamical regimes (Fig. 4*C*). We argue that the superior ability of heterogeneous networks for function generation could be explained by two factors.

First, networks with higher spike threshold heterogeneity exhibited higher-dimensional evoked dynamics in all three dynamical regimes (Fig. 4*D*). This monotonic relationship between neural heterogeneity and the dimensionality of network dynamics is related to the fact that extrinsic stimulation leads to stronger synchronization between neurons in homogeneous networks (*SI Appendix*, Figs. S7–S9). Strong synchronization creates periods during which a large fraction of the neurons in the recurrent network are in a refractory state and cannot contribute to function generation.

Second, networks with higher heterogeneity showed less trial-to-trial variability in their input-evoked dynamics. To quantify this effect, we calculated a kernel matrix that captures the function generation capabilities of a linear readout of the network, and quantified its variance q across trials (see *SI Appendix* for details). Since the dynamics of the networks simulated here are deterministic, different trials can only lead to different outcomes because of differences in the initial state of the network. The kernel variance q reflects how strongly the function generation capabilities of a network depend on its intrinsic initial state. As shown in Fig. 4*E*, heterogeneous networks expressed less kernel variance than homogeneous networks. This suggests that heterogeneous networks are more robust to fluctuations in their intrinsic dynamics and are thus more reliable function generators than homogeneous networks.

Fig. 4*C* shows that networks in a synchronized regime have typically worse function generation capabilities than networks in asynchronous regimes. This result is to be expected: Theories of brain oscillations posit that the ability of an extrinsic input to generate a network response depends on whether the network is in an active (up) or inactive (down) state within an oscillation cycle when the input arrives (57, 58). In other words, stimulus-evoked dynamics depend on the intrinsic state of synchronized network at the time of stimulation. Since networks were trained to find input–output transformations that are independent of the intrinsic state, synchronous networks have to find an input–output mapping that is independent of the oscillatory phase at which the input arrived. This explains why synchronous networks show decreased performance in this task compared to asynchronous networks. Interestingly, this effect is relatively small compared to the effect of heterogeneity on function generation performance. We note that a heterogeneous network in a synchronous regime can achieve a function generation performance comparable to that of a more homogeneous network in an asynchronous active regime.

**Fig. 5.**
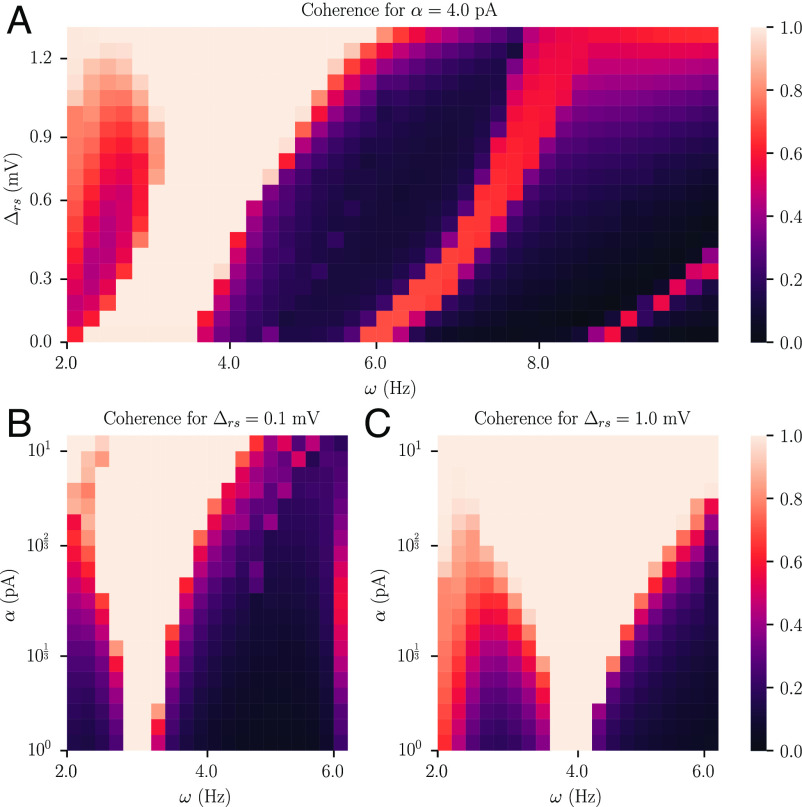
Spike threshold heterogeneity affects the entrainment properties of a network of spiking neurons. (*A*) Coherence between a sinusoidal driving signal of strength α=4 pA and the fluctuations in the average firing activity r of the network as a function of the driving frequency ω and the neural heterogeneity $\Delta_{\mathrm{rs}}$. (*B*) Coherence between a sinusoidal driving signal and the fluctuations of the average firing activity r for an homogeneous network ($\Delta_{\mathrm{rs}}=0.1$ mV) as a function of the driving frequency ω and driving strength α. (*C*) Same as *B* but for a heterogeneous network ($\Delta_{\mathrm{rs}}=1.0$ mV).

To gain further insight into this result, we examined the effect of neural heterogeneity on the ease of entrainment for a network of regular-spiking neurons. Specifically, we studied the mean-field Eqs. **6**–**9** under periodic forcing, and quantified the phase coherence between the periodic forcing signal and the fluctuations in the average firing activity of the network. We found that heterogeneous networks could be entrained to oscillate over a larger range of forcing frequencies ω than homogeneous networks (Fig. 5*A*); the effect holds for various strengths α of the periodic forcing signal (Fig. 5 *B* and *C*). This increased sensitivity to periodic forcing explains why heterogeneous oscillating networks are capable of reliable function generation: Their comparative ease of entrainment implies that a large number of neurons will be able to respond to the input signal.

## Discussion

Structural heterogeneity has a profound impact on the emergent dynamics of complex dynamical systems (59–62), as has been demonstrated in scenarios as diverse as coupling in chemical oscillators (63), climate policy (64), cellular disease spreading (65), and the achievement of herd immunity (66).

Here, we have shown that within-type heterogeneity in networks of coupled spiking neurons has a similarly strong impact on neural population dynamics, with important consequences for the types of computations those networks can carry out. While previous work has emphasized that within-type heterogeneity is an important mechanism for desynchronizing neural dynamics in the brain (22, 23, 67), we found that within-type heterogeneity is an important property, one that controls the functions of neural networks to gate, encode, and transform signals. Our findings inform two hypotheses about the role that within-type heterogeneity plays in shaping the computational strategies employed by the brain, presented below. Both hypotheses assume that it is mostly the excitatory neurons that encode and transform behaviorally relevant information in the brain, whereas inhibitory neurons gate the information flow across networks of excitatory neurons (11).

### Hypothesis I: The Heterogeneity of Inhibitory Interneurons Serves to Adjust Their Mechanism for Gating Excitatory Neural Signals.

The gating of excitatory signal flow has been suggested as one of the main functions of inhibitory neurons (68–70). Based on our results, we hypothesize that the heterogeneity of inhibitory neurons shapes this gating function. This hypothesis is based on our finding that the level of heterogeneity of the inhibitory interneuron population determines whether it acts as a phase resetting mechanism or as mere shift in the global input to excitatory neurons.

Heterogeneous inhibitory interneurons effectively act as a global input that regulates the level of activation of local excitatory populations. In this role, they induce the same phase transitions that can be caused by extrinsic inputs to the excitatory population. Extrinsic inputs that activate heterogeneous inhibitory populations can cause transitions between up-states and down-states in the excitatory population and thus contribute, for instance, to short-term memory functions of the network (51, 71). Alternatively, extrinsic inputs to the inhibitory population could move the excitatory population in and out of an oscillatory regime and thus cause changes in the communication pathways within the brain (57, 58, 72).

In contrast, homogeneous inhibitory interneurons play a phase resetting role, since activating these neurons leads to a synchronized response that resets the entire network state. This could serve as a mechanism to control the initial response of the excitatory population to an extrinsic input, or to flush the network memory (73–75). At high levels of interneuron homogeneity, neural networks become prone to periodic spike synchronization, leading to high-frequency oscillations. In the cortex, the generation of these high-frequency oscillations has been linked to the processing and transmission of sensory information (76). Excitatory-inhibitory local circuits, such as the networks of regular-spiking and fast-spiking neurons studied here, are suggested to synchronize at these high frequencies to transmit information across distributed brain networks (77). The oscillatory properties of such circuits have recently been shown to depend on levels of neural heterogeneity (78), thus confirming our results on the critical role that inhibitory interneuron heterogeneity plays in spike synchronization. At larger scale, homogeneous interneurons have also been related to neurological disorders, as it has been suggested that a minimum degree of heterogeneity in inhibitory neural populations is required to prevent pathologically synchronized brain states (22, 67).

### Hypothesis II: The Heterogeneity of Excitatory Neurons Serves to Optimize Their Capacities to Encode and Transform Signals.

This hypothesis is an extension of recent studies that found that the performance of neural networks on various classification and function approximation tasks could be improved by allowing for heterogeneous neuron parameters (79–81). Our work extends these findings by demonstrating that whether more homogeneous or more heterogeneous networks are computationally beneficial differs between encoding and signal transformation tasks.

First, we consider neural networks in asynchronous, multistable regimes that are dynamically well equipped for memory tasks (82, 83). We found that homogeneous networks express larger multistable regimes than heterogeneous networks (Figs. 2*A* and 3*A*). Furthermore, the homogeneous networks were less susceptible to the diffusion of localized activity and thus more capable of maintaining different stable states in different parts of the network. Our findings complement previous work that found that heterogeneity in the strength of synaptic coupling in ring networks leads to similar increases in the diffusion of activity (84).

Together, these results suggest that homogeneous networks have a greater memory capacity than heterogeneous networks. This property has direct implications for biological networks where information about location and body position is encoded by spatially confined bumps of neural activity (11, 85, 86). Our hypothesis implies that reliable encoding of information via a spatial activity “bump” necessitates that the excitatory neurons in the network are relatively homogeneous. This network configuration may be achieved by a combination of intrinsic neural homogeneity and homeostatic mechanisms such as short-term synaptic plasticity that can compensate for intrinsic heterogeneities (87, 88).

Second, neural networks in an oscillatory regime have been proposed to play a crucial role in long-range information transmission and integration in the brain (57, 58, 72). It has been argued that oscillating networks can gate information flow because they only respond well to extrinsic input in the up-phase of their intrinsic oscillation cycle while they are barely responsive during their down-phase (57). Our results suggest that this is true only for homogeneous networks and that neural heterogeneity makes the network response more robust to phase differences between intrinsic oscillation and extrinsic input. When in their oscillatory regime, heterogeneous networks do not require phase synchronization with their input sources to reliably respond to them. Hence, neural heterogeneity is a property that allows stable communication and computation in neural oscillators, despite unstable phase relationships.

Moreover, heterogeneous networks generally express a better function generation capacity than homogeneous networks. These findings are directly related to the high dimensionality of the intrinsic dynamics of heterogeneous networks, which reflects the fact that heterogeneous networks, whether oscillating or not, include fewer neurons with high spike train correlations than homogeneous networks.

These modeling results provide insight into previous experimental findings. In electric fish, it has been reported that neural heterogeneity is associated with an improved ability to discriminate between different courtship signals (12). Recordings from olfactory bulb cells in mice have provided evidence that neural heterogeneity might contribute to the diversity of receptive fields inside a neural population (13, 89). Our hypothesis implies that these findings are manifestations of excitatory networks operating in a regime where heterogeneity provides a richer repertoire of collective dynamics that allow for more reliable transformations of the inputs to the network than the neural dynamics of its homogeneous counterparts.

### Generalization of Our Results to Other Scenarios.

In this work, we have studied networks of coupled spiking neurons with heterogeneous spike thresholds sampled from a Lorentzian distribution. We chose to consider heterogeneity in spike thresholds because they are a measurable property that reflects electrophysiological aspects of a cell that determine how input currents are translated into firing rates. Spike threshold heterogeneity is thus a viable means to induce firing rate heterogeneity. We chose a Lorentzian distribution to characterize the variability of spike thresholds because the mathematical properties of this distribution allowed to derive the mean-field Eqs. **6**–**9** analytically. These mean-field equations were pivotal in obtaining the results presented here, since they allowed us to relate the degree of spike threshold heterogeneity to the function of a neural network by studying changes in the mean-field dynamics of the network. We now discuss how well our results generalize to i) parameters other than the spike threshold and ii) non-Lorentzian probability distributions.

Regarding (i), we would like to emphasize that the Izhikevich neuron model behaves similar to a quadratic integrate-and-fire neuron with spike-frequency adaptation (48). A change of variables relates quadratic integrate-and-fire neurons to non-linear phase oscillators (36). From the perspective of a non-linear phase oscillator, a distribution over any Izhikevich neuron parameter translates into a distribution of the intrinsic frequency of the oscillator. Thus, we expect the introduction of heterogeneities in different Izhikevich neuron parameters to have qualitatively similar effects on the network dynamics and function.

Regarding (ii), heavy tails that characterize the Lorentzian distribution raise the question whether our results are in part driven by large numbers of extreme spike thresholds. We addressed this concern by using a truncated Lorentzian distribution for all our simulations of spiking neural network; this strongly limited the number of extreme spike thresholds in the network. For a detailed analysis of how this truncation affects the accuracy of the mean-field equations in describing the dynamics of the simulated networks of spiking neurons, see ref. 90. In addition, we performed a detailed comparison of the spiking network dynamics obtained from simulated networks using Lorentzian vs. Gaussian distributions of spike thresholds (Fig. 1). We found that these networks produce comparable dynamic regimes, a result that adds to previous studies analyzing the potentially different effects of Gaussian vs. Lorentzian parameter distributions on the collective dynamics of neural networks (36, 91, 92). Despite reporting quantitative differences between the dynamics of neural networks with Gaussian vs. Lorentzian heterogeneity distributions, these studies failed to find qualitative differences, such as a dynamic regime that can be found for one type of distribution but not for the other. However, it is still unclear whether this robustness would still hold for spike threshold distributions that are non-symmetric or multi-modal. We thus call on future studies to generalize the results presented in this work to other types of distributions for characterizing the heterogeneity of neural properties.

### Conclusion.

In conclusion, we have proposed and provided evidence for considering neural heterogeneity as a crucial mechanism for shaping neural computation. Within-type heterogeneity controls the gating functions of inhibitory interneurons as well as the capacities of excitatory neurons to encode and transform signals. Homogeneous excitatory networks can be leveraged for reliable information encoding via localized states of persistent activity, whereas heterogeneous networks provide robust and flexible information processing capacities in regimes of oscillatory dynamics. The control of heterogeneity thus allows the brain to optimize the local neural circuits for particular tasks.

## Materials and Methods

### A. Model Equations and Parameters.

We studied networks of Izhikevich neurons of the form Eqs. **1**–**4**. The state of the network is defined by the membrane potentials $v_{i}$ of all neurons {i}, a global recovery variable u, and a global post-synaptic activation term s. A neuron generates a spike when its membrane potential reaches a cutoff value $v_{p}$. At the time point t when $v_{i}\left(t\right)=v_{p}$, a spike is counted and the membrane potential $v_{i}$ is reset to a potential $v_{0}$. Hence, Eq. **4** represents the average firing activity across all neurons in the network; here δ is the Dirac delta function and $t_{j}^{k}$ is the time point when the k-th spike of the j-th neuron was generated. Since the average firing rate r drives the synaptic input s that each neuron in the population receives, Eqs. **1**–**4** represent an all-to-all coupled system. Eq. **3** defines the synaptic input as a low-pass filtering of all spikes arriving at a neuron; the exponential kernel of this filter reflects the slow synaptic integration of the input across the dendritic tree of each neuron (41). The average firing rate r also affects u, a global recovery variable that accounts for spike frequency adaptation and subthreshold oscillations of membrane potentials. Whereas the original IK model used neuron-specific recovery variables $u_{i}$ (39), we approximate their effects via a global recovery variable u shared across neurons. As we show in ref. 90, this approximation captures quite well the macroscopic dynamics of networks with neuron-specific recovery variables $u_{i}$. This approximation, together with the adoption of a Lorentzian assumption for the probability distribution of the spike thresholds $v_{\theta }$, allows for an analytic derivation of the mean-field Eqs. **6**–**9**, which self-consistently capture the macroscopic dynamics of the spiking neural network described by Eqs. **1**–**4**. We provide a detailed derivation of the mean-field equations in *SI Appendix*.

The set Eqs. **1**–**4** models a single population of all-to-all coupled neurons that interact uniformly via a single synapse type of strength J with synaptic filtering time constant $\tau_{s}$. To generalize this model to multiple populations that interact via distinct synapse types, it suffices to add additional synaptic currents of the form $I^{m}=g^{m}s_{i}^{m}\left(E^{m}-v_{i}\right)$ to the right-hand side of Eq. **1**; the superscript m identifies a particular synapse type with maximum conductance $g^{m}$, reversal potential $E^{m}$ and synaptic current $s_{i}^{m}$. Each synaptic current $s_{i}^{m}$ may be governed by a distinct synaptic low-pass filtering:

[10] $$\begin{array}{c} \tau_{s}^{m}{\dot{s}}_{i}^{m}=-s_{i}^{m}+\tau_{s}^{m}\sum_{j=1}^{N}J_{\mathrm{ij}}^{m}\sum_{k\backslash t_{j}^{k}\leq t}\delta \left(t-t_{j}^{k}\right), \end{array}$$

where $J_{\mathrm{ij}}^{m}$ is a coupling strength specific to the synapse from neuron j to neuron i via synapse type m. We used this definition of the synaptic input to study networks of interacting regular-spiking (excitatory) and fast-spiking (inhibitory) neurons with sparse coupling. Furthermore, all SNN simulations reported in this study are based on networks with sparse, random coupling, i.e., where each synapse in the network has a strength $J_{\mathrm{ij}}$ as in Eq. **10**. This choice allowed us to compare our mean-field predictions, obtained under the assumption of all-to-all coupling, to the dynamics of more realistic SNNs.

The full equations for multi-population excitatory-inhibitory Izhikevich neuron networks can be found in our previous work (90, 93). The default parameters used for regular-spiking, fast-spiking, and low-threshold-spiking neurons are provided in Tables 1–3, respectively. Furthermore, Table 4 provides the coupling parameters for the two-population model consisting of coupled regular-spiking and fast-spiking neurons. While we have found similar results for a number of different coupling parameter sets (see *SI Appendix*, Fig. S13 for results obtained for two additional sets of coupling parameters), we chose the particular set of coupling parameters in Table 4 to reflect i) the larger number of excitatory vs. inhibitory neurons in the cortex (5) and ii) the balanced strength of excitatory and inhibitory synaptic inputs to cortical neurons (49, 94).

**Table 1.** Regular-spiking neuron parameters

Parameter	Value	Parameter	Value
C	100pF	k	0.7nS/mV
$v_{r}$	−60mV	${\overset{¯}{v}}_{\theta }$	−40mV
g	1nS	E	0mV
$\tau_{u}$	33.33ms	$\tau_{s}$	6.0ms
κ	10pA	b	−2.0nS
J	15	$\Delta_{v}$	0.5mV

**Table 2.** Fast-spiking neuron parameters

Parameter	Value	Parameter	Value
C	20pF	k	1.0nS/mV
$v_{r}$	−55mV	${\overset{¯}{v}}_{\theta }$	−40mV
g	1nS	E	−65mV
$\tau_{u}$	5.0ms	$\tau_{s}$	8.0ms
κ	0pA	b	0.025nS
J	5	$\Delta_{v}$	1mV

### B. Numerical Simulation and Analysis of the Dynamics of Spiking Neural Networks.

Network dynamics were obtained via numerical solutions to the initial value problem. For all model dynamics (spiking neural network dynamics as well as mean-field model dynamics), we used the simple Euler method with an integration step-size of 0.01 ms to solve the initial value problem. To obtain the mean-field model and spiking neural network dynamics, we used the open-source softwares PyRates 1.0.4 ( 95) and RectiPy 0.12.0 ( 96), respectively. Scripts to reproduce our results are available at Zenodo ( 97).

Spiking neural network dynamics were computed for networks of N = 2,000 neurons, if not mentioned otherwise. The spike threshold of each neuron was randomly sampled from a truncated Lorentzian. The distribution was truncated such that a) the minimum spike threshold was larger than the resting membrane potential and b) the distribution was symmetric (see ref. 90 for details on the truncation and the sampling of the truncated distribution). For sparse synaptic coupling, we used a coupling probability of p=20%. Each neuron received pN synaptic inputs through couplings of equal strength, from randomly sampled source neurons.

In ring networks, we randomly sampled connections from a spatial connectivity distribution with probability density function $\rho \left(x,y\right)={c|x-y|}^{-d}$, where y and x are the location indices of the source and target neurons, respectively, and c is a normalization constant to ensure that ∫ρ(x,y)dy=1 for all x. The parameter d determines how quickly the connectivity distribution falls of with the distance between neurons. We used d=1.5 and d=0.75 to obtain the results in Figs. 3 and 4, respectively. To obtain the results in Fig. 2, we used a purely random network (no ring structure); this corresponds to d=0.

To ensure that our results are independent of the random sampling of network connections, we repeated the network simulations for Figs. 3 and 4 10 times for each set of network parameters, using a different randomly sampled set of coupling strengths each time. We show results averaged across the ten repetitions as well as individual trials in Figs. 3 and 4.

**Table 3.** Low-threshold-spiking neuron parameters

Parameter	Value	Parameter	Value
C	100pF	k	1.0nS/mV
$v_{r}$	−56mV	${\overset{¯}{v}}_{\theta }$	−42mV
g	1nS	E	−65mV
$\tau_{u}$	33.33ms	$\tau_{s}$	8.0ms
κ	20pA	b	8.0nS
J	5	$\Delta_{v}$	1mV

**Table 4.** Coupling strengths for the two-population model

Parameter	Value	Parameter	Value
$J_{r,r}$	16	$J_{r,f}$	16
$J_{f,f}$	4	$J_{f,r}$	4

To obtain the bifurcation diagrams depicted in Figs. 1 and 2, we employed numerical parameter continuation and bifurcation analysis using the open-source software PyCoBi (98), which is available at Zenodo (99). This software is based on the well-known Fortran software Auto-07p (100), which implements state-of-the-art methods to continue known solutions of differential equation systems and automatically detect special solution types in up to three parameters (101, 102). Additionally, we located bifurcation points in the SNN dynamics for Fig. 1 based on numerical simulations with a slow ramp in the input current I(t); this procedure ensured that the network was slowly pushed toward and over the bifurcation points of interest. To detect fold bifurcations, we detected time points T where the average firing rate in the population crossed the threshold $r_{T}=10$ Hz, chosen to mark the transition from a quiescent to a persistent spiking regime (see *SI Appendix*, Fig. S10 for an example). To detect Andronov–Hopf bifurcations, we identified oscillatory amplitudes in the fluctuations of the average firing rate that spanned 10 Hz from through to peak (see *SI Appendix*, Figs. S11 and S12 for examples). If at least 5 subsequent cycles of these oscillatory patterns were detected, that value of the input current I was marked as corresponding to an oscillatory regime. The start and end points of the range of values of I corresponding to the oscillatory regime were identified as Hopf bifurcation points. A similar procedure was used in ref. 90.

### C. Analysis of Persistent Activity in Spiking Neural Networks.

Our aim in Fig. 3 was to identify regions of a ring network that expressed bump activity, i.e., a state of persistent activity after we applied extrinsic stimulation to a small section of the network. To look for persistence, we recorded the spiking activity of each neuron in the network for a time interval of duration τ = 3,000 ms (Fig. 3 *B* and *C*) at a sampling rate of 10 samples per ms (10 KHz). We applied low-pass filtering to the spike train by convolving the time series with a Gaussian kernel of width σ=200 samples (20 ms), to obtain a slowly varying firing rate that characterized the activity of individual neurons. Finally, we calculated the average firing rate of each neuron during the final 200 ms of the recording period and normalized the firing rates by the maximum firing rate observed during these final 200 ms of the recording period. We plotted the normalized firing rates for all neurons to identify the network regions that displayed persistent activity. Fig. 3 *D* and *E* show the persistent activity averaged over ten different network realizations; *SI Appendix*, Fig. S6 shows the persistent activity of different example networks. We then quantified the spread of persistent activity by computing the root mean squared error between the distribution of persistent activity across the network and the extrinsic input distribution, where the latter is simply a step function that is 1 at each location in the network where extrinsic stimulation was applied and 0 elsewhere. We also calculated the average activity outside the initial bump, defined as the average of the firing activity across all locations in the network where no extrinsic input was applied.

### D. Analysis of Function Generation Capabilities in Spiking Neural Networks.

Our aim in Fig. 4 was to quantify the capability of the recurrent neural network to generate arbitrary time-dependent functions after a short pulse was delivered as an extrinsic input. We used 50 distinct onset times for the extrinsic stimulation. For networks in the oscillatory regime, the onset times were equally spaced across a single cycle of the intrinsic oscillation ($I_{\mathrm{rs}}=55$ pA). For networks in an asynchronous regime, the onset times were equally spaced across a time period of 250 ms. We then simulated the network dynamics for an initial period of ≈ 2,000 ms (we varied this duration slightly in oscillating networks to account for differences in the intrinsic oscillation frequency) and used the final state of the network as an initial condition (in the oscillatory regime, this final state corresponded to a trough within the intrinsic oscillatory cycle). For each onset time of the extrinsic pulse input, we simulated the network dynamics until the input onset plus an additional period of ≈250 ms.

Next, we quantified the function generation capacities of the network based on its dynamics after the extrinsic stimulation. A data matrix X of dimension N×T describes the collected dynamics the N neurons in the network during T time points. We asked about the capacity of the network to generate arbitrary functions y(t), 0≤t≤T, via a linear readout:

[11] $$\begin{array}{c} y\approx wX, \end{array}$$

where w is an N-dimensional vector of readout weights. This scenario is known as reservoir computing (55): a recurrent neural network is viewed as a dynamical system that can leverage its high-dimensional state space to perform meaningful, linearly separable transformations on its inputs. The set of readout weights can be obtained analytically:

[12] $$\begin{array}{c} w=yX^{T}{\left(XX^{T}+\gamma I\right)}^{-1}, \end{array}$$

where I is the N×N identity matrix and γ corresponds to the L2 regularization strength used when solving (Eq. **11**) via ridge regression. We obtained the readout weights for two distinct target signals $y_{1}\left(t\right)$ and $y_{2}\left(t\right)$ via (Eq. **12**). The first target signal was chosen as a combination of two sine functions $y_{1}\left(t\right)=\sin\left(2\pi \omega_{1}t\right)\sin\left(2\pi \omega_{2}t\right)$, with frequencies $\omega_{1}=5$ Hz and $\omega_{2}=20$ Hz. The second target signal was chosen as the convolution of a Dirac delta function centered at $t^{*}=150$ ms with a Gaussian kernel of width σ=15 ms. The readout weights for these two target functions were calculated based on the network responses to 40 different stimulation onsets. Once the readout weights were obtained, we computed the network responses to 10 additional, randomly chosen stimulation onsets to obtain the function generation performance on test data, as follows. For each test stimulation onset, we collected the network response $X_{\mathrm{test}}$ and used the trained readout weights w to generate the response from the network dynamics via

[13] $$\begin{array}{c} \hat{y}=wX_{\mathrm{test}}. \end{array}$$

We then calculated the mean-squared error between the actual response $\hat{y}$-pagination and the target response y, and averaged this error over all 10 test stimulation onsets to quantify the capacity of the network to generate a specific target function. We provide examples for the function generation performance of homogeneous and heterogeneous networks on $y_{1}\left(t\right)$ and $y_{2}\left(t\right)$ as well as visualizations of the corresponding network dynamics in *SI Appendix*, Figs. S7–S9.

As an additional measure of function generating capacities, we estimated the dimensionality d of the network dynamics using the participation ratio (103, 104),

[14] $$\begin{array}{c} d={\left(\sum_{i}\lambda_{i}\right)}^{2}/\sum_{i}\lambda_{i}^{2}, \end{array}$$

where $\lambda_{i}$ are the eigenvalues of the covariance matrix $XX^{T}$. We found this to be a good aggregate measure that explains the differences in the function generation capacities of heterogeneous vs. homogeneous networks (Fig.4).

Finally, we calculated the variance q of the network’s function generation capacity across different stimulation onsets. We used the network response kernel K, a T×T matrix which yields information about the general function generation capacities of a network, independent of a particular target function (54, 105). The network response variance Q quantifies the trial-by-trial difference of the kernel response K: $Q_{\mathrm{ij}}=\;\text{diff}\left(K_{\mathrm{ij}}\right)$, where diff yields the average difference of K across training trials. We then compute q as:

[15] $$\begin{array}{c} q=\sum_{i}\sum_{j}Q_{\mathrm{ij}}^{2}, \end{array}$$

A larger q indicates a stronger dependence of the network response to an extrinsic stimulation on the intrinsic state of the network at the time of stimulation. We provide a detailed definition of the network response kernel K and the network response variance Q in *SI Appendix*.

## Supplementary Material

Appendix 01 (PDF)

## Data Availability

There are no data underlying this work.
