# Supplementary material for: Neural heterogeneity controls computations in spiking neural networks
Source: Proc Natl Acad Sci U S A. 2024 Jan 10;121(3):e2311885121. doi: 10.1073/pnas.2311885121 (PMC10801870; doi:10.1073/pnas.2311885121)
Supplement: Supplementary file 1 — Appendix 01 (PDF) [file pnas.2311885121.sapp.pdf]

Supporting Information for

Neural Heterogeneity Controls Computations in Spiking Neural Networks

Richard Gast, Sara A. Solla, Ann Kennedy

Richard Gast.
E-mail: richard.gast@northwestern.edu

This PDF file includes:

Supporting text
Figs. S1 to S13
SI References

Supporting Information Text

Derivation of the mean-field equations

In this section, we provide a detailed derivation for the set of mean-field equations that self-consistently capture the macroscopic dynamics of a network of coupled Izhikevich (IK) neurons of the form

$$C\dot{v}_i = k(v_i - v_r)(v_i - v_{\theta,i}) - u + I(t) + gs(E - v_i), \quad [1]$$

$$\tau_u \dot{u} = -u + b(-v_r + \frac{1}{N}\sum_{j=1}^{N} v_j) + \tau_u \kappa r, \quad [2]$$

$$\tau_s \dot{s} = -s + J\tau_s r, \quad [3]$$

$$r(t) = \frac{1}{N}\sum_{j=1}^{N} \sum_{k \setminus t_j^k \leq t} \delta(t - t_j^k). \quad [4]$$

The state of the network is thus defined by the membrane potentials v_i of all neurons i, a global recovery variable u, and a global synaptic activation term s. The average spike rate across all neurons is defined by $r(t)$. A neuron spikes when $v_i \geq v_p$, where v_p is the peak membrane potential. In this event, a spike is counted and v_i is reset to the reset potential v_0. The synaptic activation s is defined as a convolution of the average spike rate $r(t)$ with an exponential activation kernel, which can be expressed as the first-order differential equation Eq. (3) with decay time constant τ_s and coupling constant J. The recovery variable u is driven by two terms. The term κr in the right-hand side of Eq. (2) incorporates spike-frequency adaptation into the neuron model, since u enters into Eq. (1) as a hyperpolarizing variable. The term scaled by b in the right-hand side of Eq. (2) couples u to the sub-threshold membrane potential fluctuations (1). Additional parameters that control the behavior of the neuron are the cell capacitance C, the leakage parameter k, the resting potential v_r, and the recovery variable time constant τ_u. Finally, the spike threshold potential $v_{\theta,i}$ controls the value of the membrane potential at which a spike is initiated. Each neuron is defined to have an individual spike threshold $v_{\theta,i}$, thus rendering neurons in the network heterogeneous.

For the derivation of the mean-field equations, we follow the Lorentzian ansatz introduced in (2), which has recently been applied to a system of abstract, dimensionless Izhikevich neurons with distributed input currents (3). Here, we adapt this approach to derive the mean-field equations for spiking neural networks with distributed spike thresholds, as given by Eq. (1)-Eq. (3). The values of $v_{\theta,i}$ in the network model are assumed to be neuron specific and drawn from a probability distribution $p(v_\theta)$ with width Δ_v and center $\bar{v}_\theta$:

$$p(v_\theta) = \frac{1}{\pi}\frac{\Delta_v}{[v_\theta - \bar{v}_\theta]^2 + \Delta_v^2}. \quad [5]$$

In the thermodynamic limit ($N \to \infty$), the state of the system Eq. (1)-Eq. (3) is given by a density function $\rho(v, t|v_\theta)$, which expresses the probability that a neuron with spike threshold v_θ has a membrane potential v at time t. Due to the conservation of neurons, the system dynamics can be expressed via a continuity equation

$$\frac{\partial}{\partial t}\rho(v,t|v_\theta) = -\frac{\partial}{\partial v}[\frac{1}{C}[k(v - v_r)(v - v_\theta) - u + I + gs(E - v)]\rho(v,t|v_\theta)]. \quad [6]$$

For an appropriate choice of order parameters, Eq. (6) can be solved to obtain a set of mean-field equations describing the macroscopic system dynamics. We apply the Lorentzian ansatz outlined in (2) to find these order parameters. We assume that $\rho(v,t|v_\theta)$ can be fully captured by a Lorentzian probability distribution with center y and width x:

$$\rho(v,t|v_\theta) = \frac{1}{\pi}\frac{x(t,v_\theta)}{[v - y(t,v_\theta)]^2 + x(t,v_\theta)^2}. \quad [7]$$

As shown in (2), these two parameters of the Lorentzian distribution can be related to the average firing rate r given by Eq. (4) and the average membrane potential $v(t) = \int_{v_\theta} \rho(v,t|v_\theta)p(v_\theta)dv_\theta$ via

$$\frac{C\pi}{k}r(t) = \int_{v_\theta} x(t,v_\theta)p(v_\theta)dv_\theta = x(t), \quad [8]$$

$$v(t) = \int_{v_\theta} y(t,v_\theta)p(v_\theta)dv_\theta = y(t). \quad [9]$$

These relationships hold under the assumption that $v_p \to \infty$ and $v_0 \to -\infty$. Using our ansatz, we plug Eq. (7) into Eq. (6) and solve for x and y. This allows us to express the system dynamics via a single complex variable $z(t,v_\theta) = x(t,v_\theta) + iy(t,v_\theta)$, which obeys

$$C\frac{\partial}{\partial t}z(t,v_\theta) = i[-kz(t,v_\theta)^2 + i(k(v_r + v_\theta) + gs)z(t,v_\theta) + kv_r v_\theta + gsE - u + I]. \quad [10]$$

To obtain the equations for $r(t)$ and $v(t)$ from Eq. (10), we have to solve the integral

$$\dot{z} = \frac{1}{C} \int_{v_\theta} \frac{\partial}{\partial t} z(t, v_\theta) p(v_\theta) dv_\theta. \quad [11]$$

This integral can be solved analytically for Eq. (5) by evaluating $\frac{\partial}{\partial t} z(t, v_\theta)$ at the single pole of the integrand in the upper or lower half of the complex plane $v_\theta = \bar{v}_\theta \pm i\Delta_v$. The choice of pole for the evaluation of $\frac{\partial}{\partial t} z(t, v_\theta)$ is based on the requirement that $x(t) = \frac{\pi C}{k} r(t) > 0 \; \forall t$. If the pole at $v_\theta = \bar{v}_\theta + i\Delta_v$ in the upper half of the complex plane is chosen, this requirement may be violated in strongly hyperpolarized states, i.e. when $v - v_r < 0$. In this case, $\frac{\partial}{\partial t} z(t, v_\theta)$ has to be evaluated at the pole in the lower half of the complex plane, $v_\theta = \bar{v}_\theta - i\Delta_v$.

Due to Eq. (8) and Eq. (9), it holds that $z(t, \bar{v}_\theta + i\Delta_v) = x(t) + iy(t) = \frac{\pi C}{k} r(t) + iv(t)$. By plugging this relationship into Eq. (11) and solving for r and v, we obtain the following set of coupled ordinary differential equations:

$$C\dot{r} = \frac{\Delta_v k^2 \sigma_v}{\pi C} \,(v - v_r) + r(k(2v - v_r - \bar{v}_\theta) - gs), \quad [12]$$

$$C\dot{v} = kv(v - v_r - \bar{v}_\theta) - \pi C r(\Delta_v \, \sigma_v + \frac{\pi C}{k} r) \quad [13]$$

$$+ kv_r\bar{v}_\theta - u + I + gs(E - v),$$

$$\tau_u \dot{u} = b(v - v_r) - u + \tau_u \kappa r, \quad [14]$$

$$\tau_s \dot{s} = -s + \tau_s J r, \quad [15]$$

where $\sigma_v = \mathrm{sign}(v - v_r)$ is 1 when $v - v_r \geq 0$ and -1 otherwise. This final set of four coupled ordinary differential equations captures the macroscopic dynamics of a single population of coupled spiking neurons as given by Eqs.(1-3).

The outlined approach can easily be extended to networks that include multiple coupled cell types. In such a scenario, the dynamics of each cell type are given by a set of equations of the form Eq. (1)-Eq. (3), except that a post-synaptic activation variable v_n is required for each synapse type n in the network. The average firing rate $r(t)$ driving a given post-synapse type as in Eq. (3) is then a sum of the firing rates of all cell types that project to the target cell type via this synapse. Finally, the sum on the right-hand side of Eq. (1) has to be updated to include a synaptic coupling term $g_n s_n (E_n - v_i)$ for every synapse type. The mean-field derivation then follows the same steps as above, resulting in a set of mean-field equations of the form Eq. (12)Eq. (15) for each cell type. As an example, the equations for a two-population model of coupled excitatory regular spiking neurons and inhibitory fast spiking neurons is provided in (4).

Comparison of Lorentzian and Gaussian spike threshold distributions

In this section, we report comparisons between Gaussian and Lorentzian spike threshold distributions as well as the resulting firing rate distributions for networks of coupled Izhikevich neurons of the form Eq. (1)-Eq. (4) with Gaussian vs. Lorentzian distributions of the spike thresholds $v_{\theta,i}$. To compare Gaussian and Lorentzian distributions of spike thresholds, we first fitted the standard deviations σ_v of Gaussian probability distributions with density function

$$q(v) = \frac{1}{\sigma_v 2\pi} \exp(-\frac{(v - \mu_v)}{2\sigma_v^2}) \quad [16]$$

for various widths Δ_v of Lorentzian probability distributions with density function Eq. (5). For each Δ_v, we fitted a σ_v such that the squared error between the sample variances was minimized. Specifically, for each candidate σ_v to a particular Δ_v, we drew $n = 10000$ random spike thresholds v_θ from each distribution using the density functions q and p. Both distributions were centered around -40 mV and truncated symmetrically at -60 mV and -20 mV. We then calculated the variance of the sampled spike thresholds via

$$\sigma^2 = \frac{1}{n-1} \sum_{i=1}^{n} (v_{\theta,i} - \mu)^2 \quad [17]$$

where μ is the sample average. We then used Brent's method to find the σ_v that minimizes the squared error between the sample variances, i.e. $(\sigma_G - \sigma_L)^2$, where σ_G and σ_L refer to the sample variances of the Gaussian and Lorentzian distribution, respectively. The results of this procedure are depicted in Fig.S1. As can be seen, we find a consistent relationship between σ_v and Δ_v that is non-linear for small Δ_v and becomes more linear as Δ_v grows.

How do these spike threshold distributions affect firing rate heterogeneity? To answer this question, we compared the firing rate distributions of Izhikevich neuron networks of the form Eq. (1)-Eq. (4) with spike thresholds sampled from Gaussian vs. Lorentzian probability distributions. We performed this comparison for both the static firing rate distributions obtained from the network equations without recurrent coupling ($J = 0$) and with recurrent coupling ($J > 0$). The firing rate distributions for networks without recurrent coupling were obtained analytically via

$$r_i = \frac{\sqrt{4k(kv_r v_{\theta,i} + gsE - u + I) - (kv_r + kv_{\theta,i} + gs)^2}}{2\pi C}, \quad [18]$$

which can be derived from Eq. (1)-Eq. (4) in the case that $J = 0$ for any neuron that receives a positive input (4). For $J > 0$, we performed a numerical simulation in a monostable regime where only a persistent spiking solution existed, to be able to calculate distributions of static, neuron-specific firing rates r_i. After we identified such a regime via the mean-field equations Eq. (12)-Eq. (15), we simulated the neuron dynamics over a time interval of 1 s. We then counted the spikes of each neuron, which directly correspond to the neuron-specific firing rate r_i, and calculated the distribution of r_i in the network. The results of this procedure are shown in Fig.S2-Fig.S4 for populations of regular spiking, fast spiking, and low-threshold spiking neurons, respectively. Across all three neuron types, we find that Gaussian and Lorentzian spike threshold distributions result in comparable spiking activity in the coupled networks, despite the fact that the firing rate distributions in the uncoupled networks are more heavy-tailed for Lorentzian distributions. Furthermore, we find that recurrent coupling in fast spiking neurons leads to firing rate distributions where only a few neurons with low spike thresholds drive the average firing rate in the population with high intrinsic firing, thus silencing the other neurons inside the population. This is comparable to the spiking data of fast spiking neurons recorded in entorhinal cortex of mice, where only a few neurons expressed consistent spiking without extrinsic stimulation, whereas the majority of neurons only expressed spiking activity during optogenetic stimulation (5).

Effects of spike threshold heterogeneity of inhibitory interneuron populations

We have shown in Fig.2 of the main manuscript that the heterogeneity of fast-spiking interneurons is an important control variable for its regulatory impact on the dynamics of excitatory neural populations in mesoscopic brain circuits. Heterogeneous inhibitory interneurons preserve the bifurcation structure of the excitatory population, whereas homogeneous inhibitory interneurons overwrite this bifurcation structure and move the system in the vicinity of highly synchronized dynamics. Here, we extend these results to a three population model that contains an additional inhibitory interneuron type: Low-threshold spiking neurons. For the full set of equations and parameters of this model, see (6).

In this model, we were able to replicate the results from Fig.2 of the main manuscript. Heterogeneous low-threshold spiking neurons preserved the bifurcation structure of the regular spiking neurons and allowed to induce the same phase transitions via extrinsic input to the low-threshold spiking population that could be induced via extrinsic input to the isolated regular spiking population (see Fig.S5B and F). This was not possible in homogeneous low-threshold spiking populations, however, where dynamic regimes of synchronized oscillations dominate and effectively overwrite the bifurcation structure of the regular spiking population (see Fig.S5D and H).

Note that these different modes of interaction between regular spiking neurons and low-threshold spiking neurons can be found for biologically realistic differences of the spike threshold heterogeneity in the low-threshold spiking population. Specifically, the different values of Δ_{lts} used in Fig. S5 correspond to the sample variances reported for low-threshold spiking neurons of rats in different cortical layers (7). The results in Fig..S5 were obtained for an intermediate level of fast-spiking neural heterogeneity ($\Delta_{fs} = 0.8$), which we fitted to sample variances reported for recordings of fast-spiking neurons in mice (8).

Examples for the Formation of Local Bumps in Ring Networks

In Fig.3 of the main manuscript, we show that homogeneous networks are on average better in maintaining states of persistent activity within a stimulated section of a ring network than heterogeneous networks. These local states of persistent activity are also known as "bumps". In Fig.3D and E, we show how the bump states on average change as a function of the stimulation width for a homogeneous and a heterogeneous network, respectively. The average was in this case taken across 10 different runs of the simulation, each with a different random ring network. Here, we provide examples of how these bump states change as a function of the stimulation width for three different heterogeneity levels: $\Delta_{rs} \in 0.2, 0.8, 1.6$. For each heterogeneity level, we depict the emerging persistent states (after stimulation) for two representative example networks (see Fig.S6). As can be seen, these examples are consistent with the averaged results depicted in Fig.3 of the main manuscript. Even for a particular random ring network, the capacity to maintain a persistent state only within the stimulated section of the network decreases with increasing heterogeneity.

Calculation of the network response kernel

For the results depicted in Fig.4 of the main manuscript, we calculated a network response kernel, which can be viewed as a measure of the generalized function generation properties of the network, independent of any target function $y(t)$ that the network is supposed to generate. Given a particular function $y(t)$, a set of readout weights can be trained via Ridge regression that approximates $y(t)$ as

$$y \approx wX, \qquad [19]$$

with linear readout weights w and an $N \times T$ matrix X containing the dynamics of N neurons over T time points. If you collect the network dynamics for M training trials with different initial conditions, yielding X_m with $m \in 1, 2, ..., M$, the problem is to find a single set of readout weights w that minimize the combined error between the approximations wX_m and y over all training trials. We can then rewrite the network response in a given trial as the sum of an average response $\bar{X}$ and a trial-specific noise term ϵ_m: $X_m = \bar{X} + \epsilon_m$. Then, the Ridge regression solution for w can be written as

$$w = y\bar{X}^T(\bar{C} + \gamma I). \quad [20]$$

where γ corresponds to the L2 regularization strength, and $\bar{C}$ is the average neural correlation matrix $C = \frac{1}{M}\sum_m X_m X_m^T$. The network response kernel can then be obtained in two steps. First, we multiply Eq. (20) with $\bar{X}$ from the right side, which yields

$$w\bar{X} = y\bar{X}^T(\bar{C} + \gamma I)^{-1}\bar{X}. \quad [21]$$

Then, since $y \approx w\bar{X}$, we multiply with y^{-1} from the left side to obtain the generalized network response kernel

$$K = \bar{X}^T(\bar{C} + \gamma I)^{-1}\bar{X}. \quad [22]$$

The closer K is to the identity matrix, the better the average function generation properties of the network, independent of which target function $y(t)$ is to be generated. It is important to keep in mind, however, that K is a kernel that was calculated as an average across training trials. Thus, the function generation performance may still fluctuate between particular trials. The amount of trial-by-trial variance of each entry of K can be calculated as

$$Q = \frac{1}{M}\sum_m [\epsilon_m(\bar{C} + \gamma I)^{-1}\bar{X}]. \quad [23]$$

Effects of spike threshold heterogeneity on function generation capacities of regular spiking neurons

In Fig.4 of the main manuscript we provide evidence that heterogeneity of excitatory neurons in recurrently coupled networks increases the function generation capacities of the network. Here, we provide further details regarding these results. Fig.S7-Fig.S9 provide detailed information regarding the network dynamics and function generation capacities of a network of regular spiking neurons in the quiescent, oscillating, and persistent activity regime, respectively. Most importantly, the comparison between left and right columns of Fig.S7-Fig.S9 confirms that heterogeneous networks outperform homogeneous networks with respect to function generation. We demonstrate this on two distinct target functions that the network was required to generate.

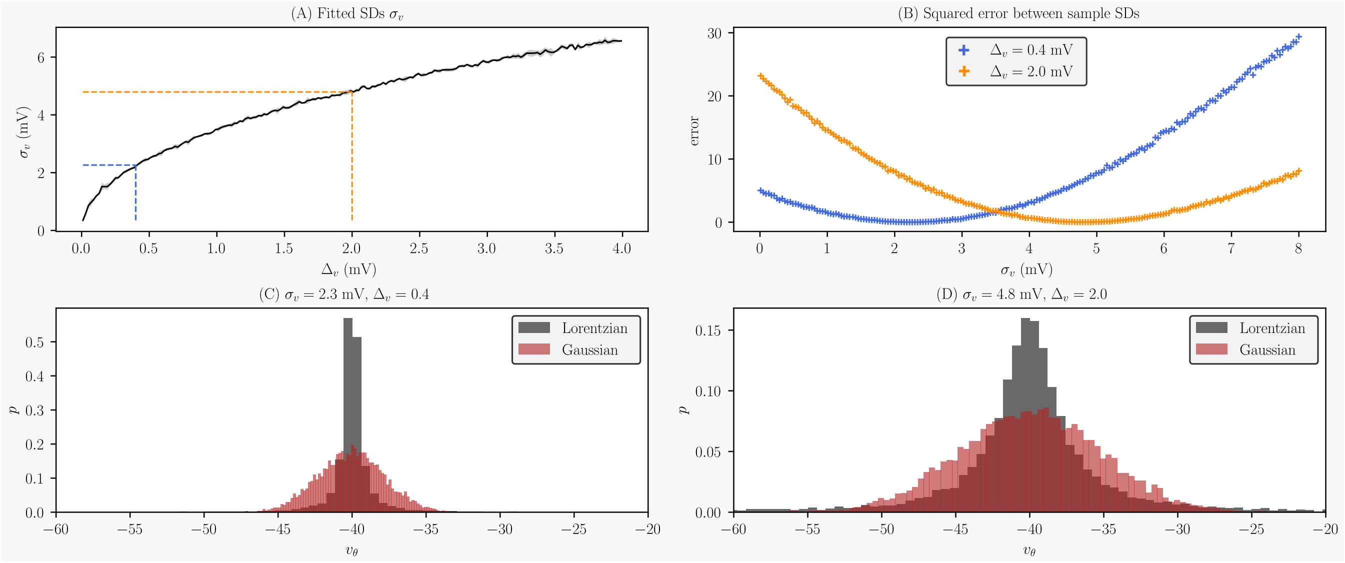

Fig. S1. Fits between Gaussian distributions and Lorentzian distributions of spike thresholds. **(A)** Standard deviations σ_v of Gaussian distributions fitted to match the sample variance of Lorentzian distributions with width Δ_v. The orange and blue dashed lines mark example fits for which the error landscape and corresponding distributions are depicted in B-D. **(B)** Squared error between the sample variance of Gaussian distributions with standard deviation σ_v and two Lorentzian distributions with width $\Delta_v = 0.4$ mV and $\Delta_v = 2.0$ mV depicted in blue and orange, respectively. **(C,D)** Examples of spike threshold distributions obtained via random sampling from Gaussian and Lorentzian distributions with σ_v and Δ_v chosen according to the blue and orange dashed lines in A.

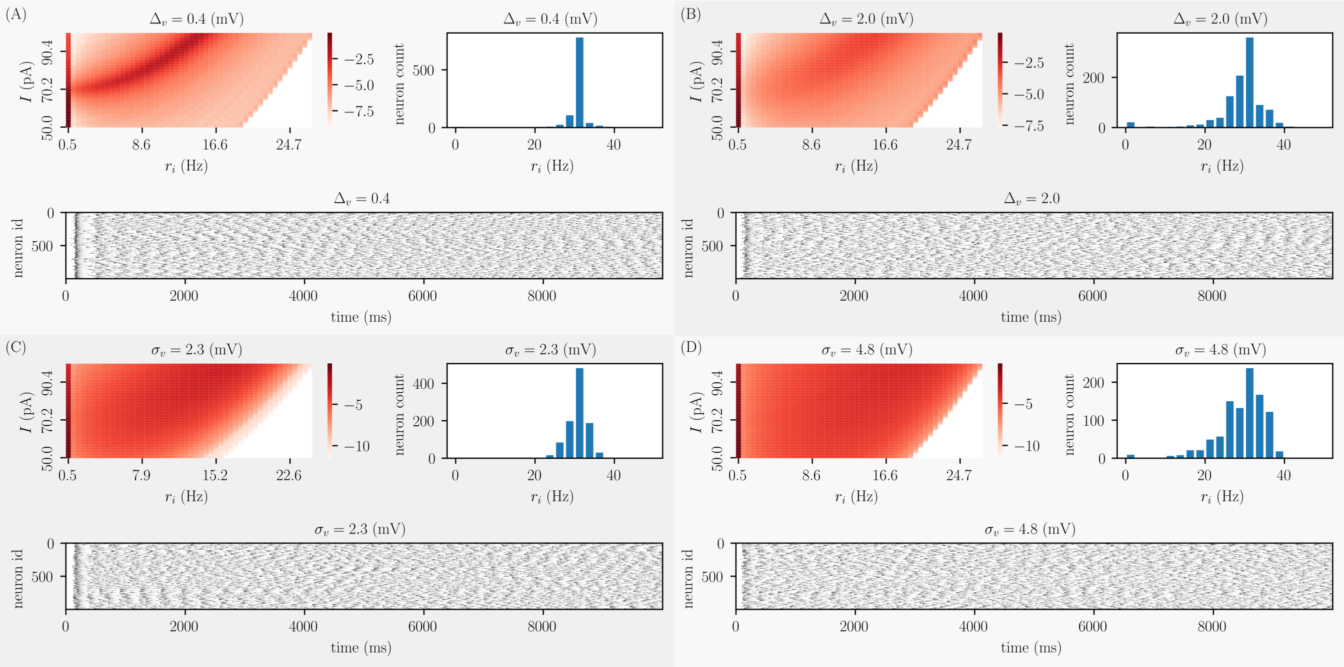

Fig. S2. Firing rate distributions of a population of regular spiking neurons. **(A,B)** Firing rate characteristics of an Izhikevich neuron population with a Lorentzian distribution of spike thresholds with $\Delta_v = 0.4$ mV and $\Delta_v = 2.0$ mV, respectively. **(C, D)** Firing rate characteristics of an Izhikevich neuron population with a Gaussian distribution of spike thresholds with $\sigma_v = 2.3$ mV and $\sigma_v = 4.8$ mV, respectively. The top rows of A-D depict firing rate distributions in the uncoupled and coupled network. On the left, static firing rate distributions for the uncoupled network are shown for various static input strengths I. The color coding represents the log probability densities, with zero probabilities being depicted in white. On the right, the firing rate distribution in the coupled network is shown for the static input $I = 50$ pA. In the bottom row, the spike raster plot corresponding to the firing rate distribution of the coupled network is shown.

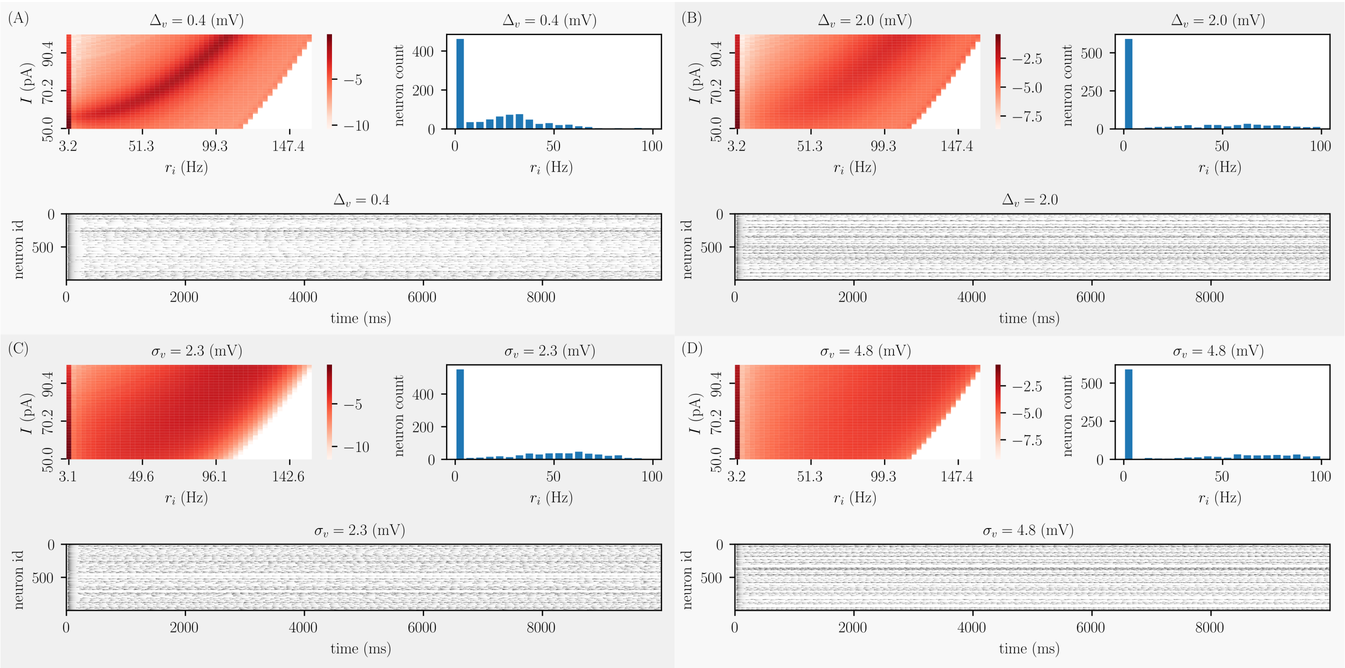

Fig. S3. Firing rate distributions of a population of fast spiking neurons. **(A,B)** Firing rate characteristics of an Izhikevich neuron population with a Lorentzian distribution of spike thresholds with $\Delta_v = 0.4$ mV and $\Delta_v = 2.0$ mV, respectively. **(C, D)** Firing rate characteristics of an Izhikevich neuron population with a Gaussian distribution of spike thresholds with $\sigma_v = 2.3$ mV and $\sigma_v = 4.8$ mV, respectively. The top rows of A-D depict firing rate distributions in the uncoupled and coupled network. On the left, static firing rate distributions for the uncoupled network are shown for various static input strengths I. The color coding represents the log probability densities, with zero probabilities being depicted in white. On the right, the firing rate distribution in the coupled network is shown for the static input $I = 70$ pA. In the bottom row, the spike raster plot corresponding to the firing rate distribution of the coupled network is shown.

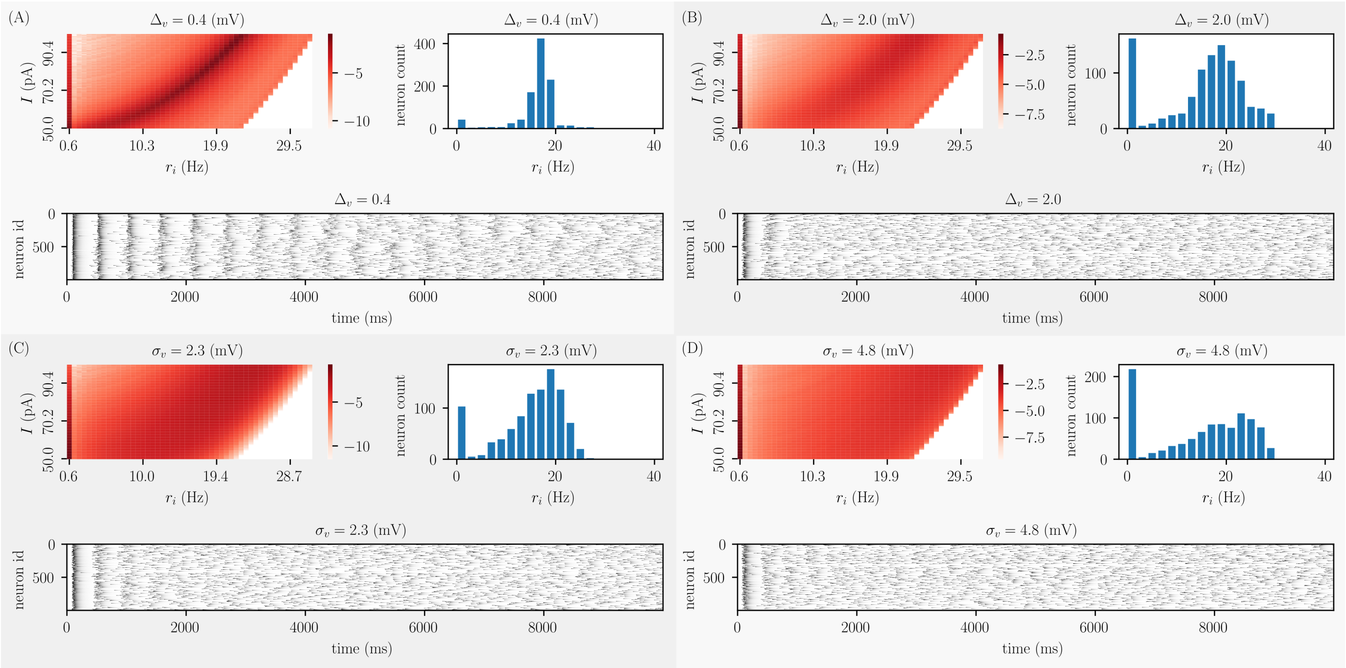

Fig. S4. Firing rate distributions of a population of low-threshold spiking neurons. **(A,B)** Firing rate characteristics of an Izhikevich neuron population with a Lorentzian distribution of spike thresholds with $\Delta_v = 0.4$ mV and $\Delta_v = 2.0$ mV, respectively. **(C, D)** Firing rate characteristics of an Izhikevich neuron population with a Gaussian distribution of spike thresholds with $\sigma_v = 2.3$ mV and $\sigma_v = 4.8$ mV, respectively. The top rows of A-D depict firing rate distributions in the uncoupled and coupled network. On the left, static firing rate distributions for the uncoupled network are shown for various static input strengths I. The color coding represents the log probability densities, with zero probabilities being depicted in white. On the right, the firing rate distribution in the coupled network is shown for the static input $I = 150$ pA. In the bottom row, the spike raster plot corresponding to the firing rate distribution of the coupled network is shown.

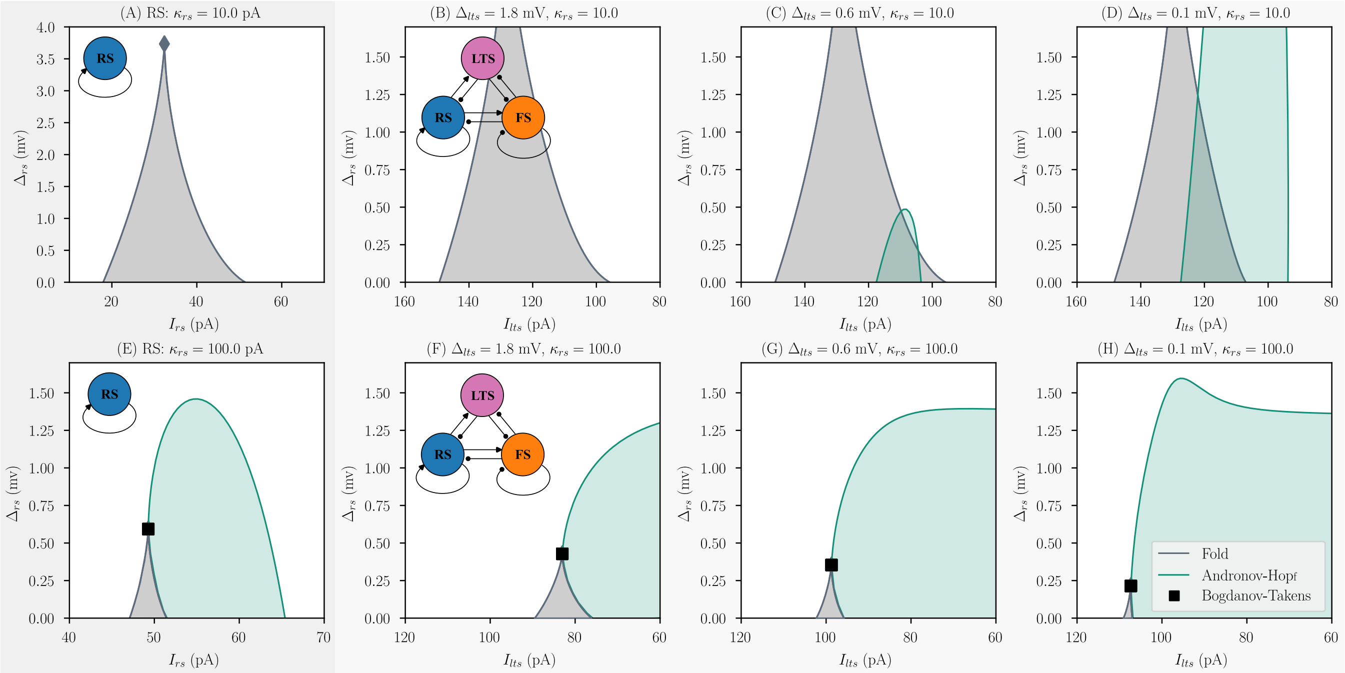

Fig. S5. Effects of spike threshold heterogeneity of low-threshold spiking interneurons in mesoscopic neural circuits. **(A, E)** Same as Fig.1A and B of the main manuscript, i.e. 2D bifurcation diagrams of a single population of regular spiking neurons for two different values of κ_{rs}. **(B-D)** 2D bifurcation diagrams of a three-population model of coupled regular spiking (RS), fast spiking (FS), and low-threshold spiking neurons (LTS). A sketch of the neural circuit is shown in B. The two bifurcation parameters are the input to the LTS population I_{lts} and the spike threshold heterogeneity of the RS population Δ_{rs}. The model was initialized such that the isolated RS population expresses a bi-stable regime ($\kappa_{rs} = 10$). B-D differ in the level of spike-threshold heterogeneity of the LTS population. Other levels of neural heterogeneity in the model were kept constant: $\Delta_{rs} = 0.4$ and $\Delta_{fs} = 0.8$. **(F-H)** Same as B-D, but the model was initialized such that the isolated RS population expresses an oscillatory regime ($\kappa_{rs} = 100$).

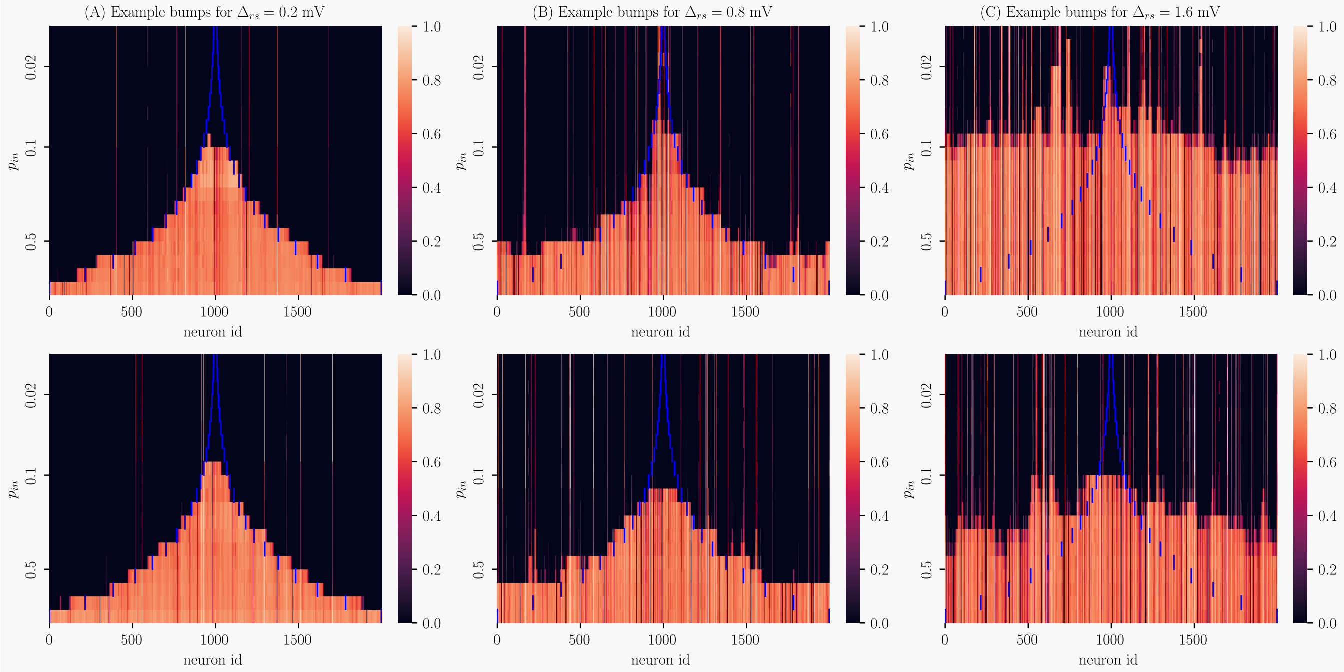

Fig. S6. Effects of spike threshold heterogeneity on local states of persistent activity. **(A-C)** Persistent (bump) activity of each neuron (x-axis) as a function of input width (y-axis.) Color-coded is the normalized neural activity, averaged over the final 200 ms of a 2000 ms window after the extrinsic stimulus was turned off. The blue horizontal lines delimit the region of the network that received extrinsic input. A-C differ in the level of spike threshold heterogeneity in the network. The top and bottom row are two distinct example networks, which differ only in the randomly sampled network connections and spike thresholds of neurons.

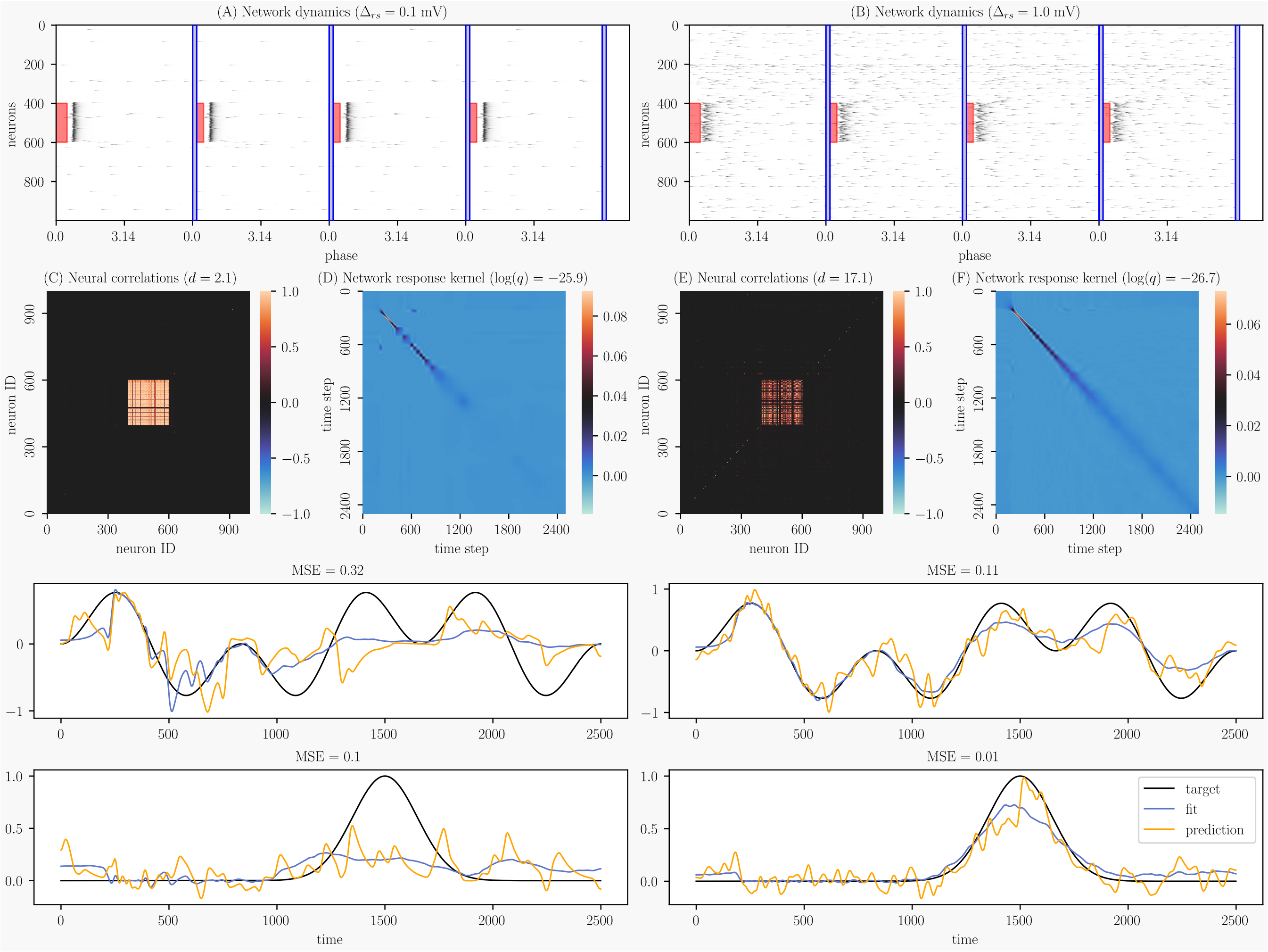

Fig. S7. Function generation capacity of asynchronous, quiescent regular spiking neurons ($I_{rs} = 45$ pA). **(A, B)** Network dynamics in response to extrinsic stimulation via short, rectangular pulses. Blue vertical bars separate independent trials. Red-shaded regions indicate both the time interval in which extrinsic stimulation was applied and the network section that was stimulated. The dynamics are shown for a homogeneous ($\Delta_{rs} = 0.1$ mV) and a heterogeneous ($\Delta_{rs} = 1.0$ mV) network. All results depicted below A vs. B correspond to the homogeneous vs. heterogeneous network, respectively. **(C, E)** Pairwise correlations of all neurons in the network, calculated across all test trials. The dimensionality of the network dynamics d was calculated based on the eigenvalue spectrum of the correlation matrix. **(D, F)** Network response kernel K. The variance in the function generation capacity q was calculated as the sum over the squared entries of the network response variance matrix Q. **(G)** Depiction of the network performance in approximating two different target functions. The black line depicts the target function, the blue line the average fit of this target function across all training trials, and the orange line depicts the function generated by the network on a particular test trial.

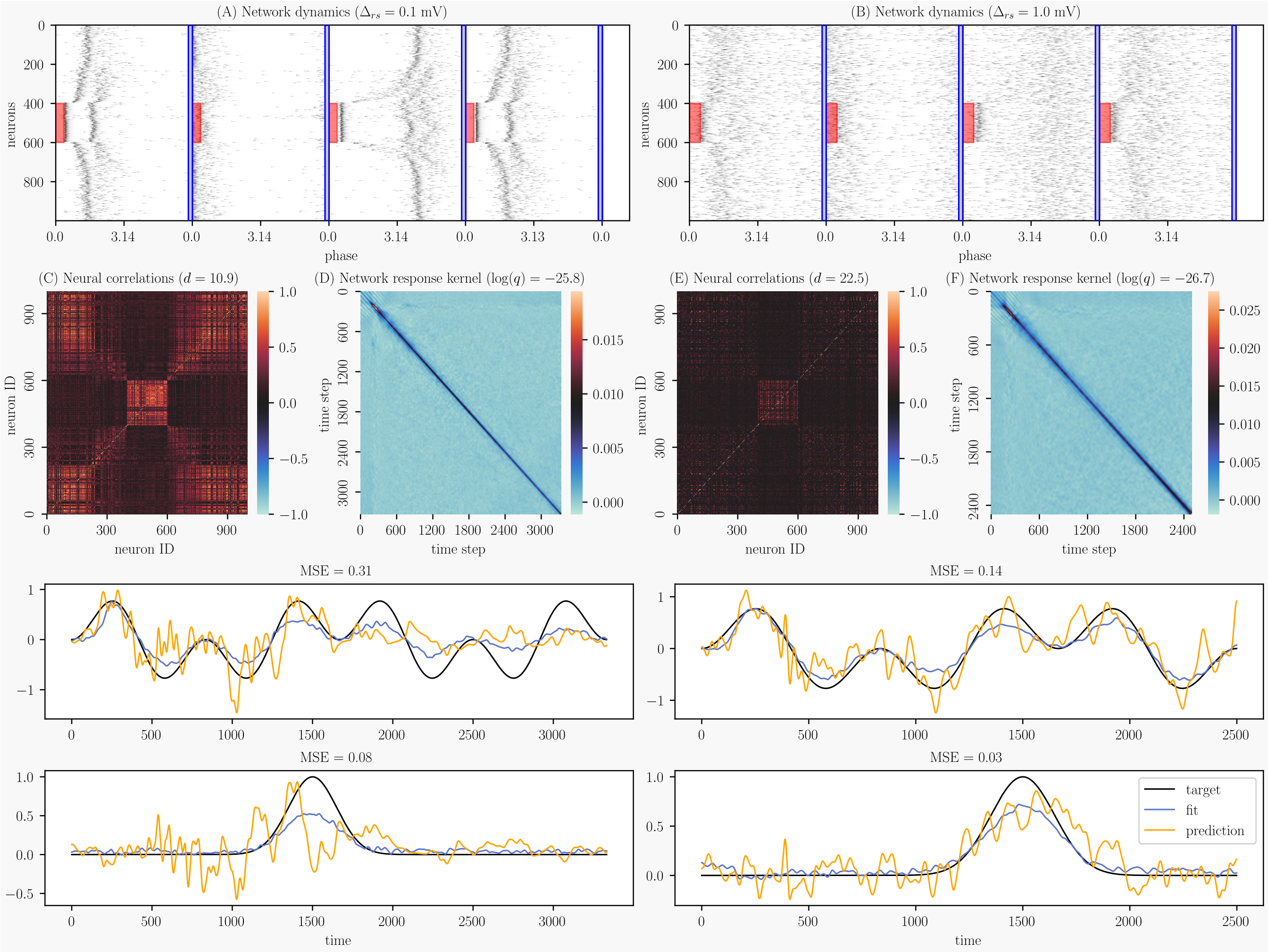

Fig. S8. Function generation capacity of synchronous, oscillating regular spiking neurons ($I_{rs} = 55$ pA). **(A, B)** Network dynamics in response to extrinsic stimulation via short, rectangular pulses. Blue vertical bars separate independent trials. Red-shaded regions indicate both the time interval in which extrinsic stimulation was applied and the network section that was stimulated. The dynamics are shown for a homogeneous ($\Delta_{rs} = 0.1$ mV) and a heterogeneous ($\Delta_{rs} = 1.0$ mV) network. All results depicted below A vs. B correspond to the homogeneous vs. heterogeneous network, respectively. **(C, E)** Pairwise correlations of all neurons in the network, calculated across all test trials. The dimensionality of the network dynamics d was calculated based on the eigenvalue spectrum of the correlation matrix. **(D, F)** Network response kernel K. The variance in the function generation capacity q was calculated as the sum over the squared entries of the network response variance matrix Q. **(G)** Depiction of the network performance in approximating two different target functions. The black line depicts the target function, the blue line the average fit of this target function across all training trials, and the orange line depicts the function generated by the network on a particular test trial.

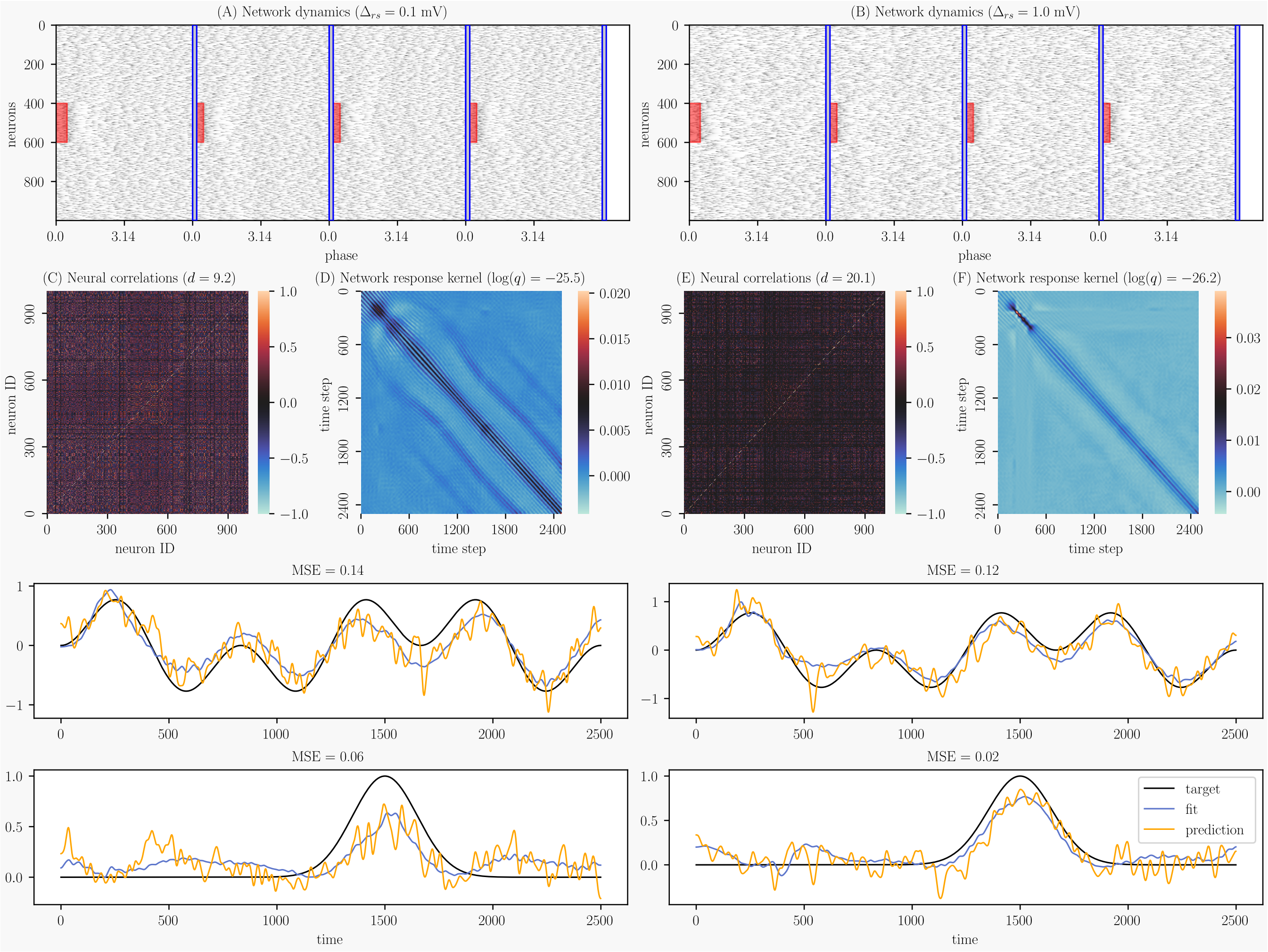

Fig. S9. Function generation capacity of regular spiking neurons in an asynchronous regime with persistent spiking activity ($I_{rs} = 70$ pA). **(A, B)** Network dynamics in response to extrinsic stimulation via short, rectangular pulses. Blue vertical bars separate independent trials. Red-shaded regions indicate both the time interval in which extrinsic stimulation was applied and the network section that was stimulated. The dynamics are shown for a homogeneous ($\Delta_{rs} = 0.1$ mV) and a heterogeneous ($\Delta_{rs} = 1.0$ mV) network. All results depicted below A vs. B correspond to the homogeneous vs. heterogeneous network, respectively. **(C, E)** Pairwise correlations of all neurons in the network, calculated across all test trials. The dimensionality of the network dynamics d was calculated based on the eigenvalue spectrum of the correlation matrix. **(D, F)** Network response kernel K. The variance in the function generation capacity q was calculated as the sum over the squared entries of the network response variance matrix Q. **(G)** Depiction of the network performance in approximating two different target functions. The black line depicts the target function, the blue line the average fit of this target function across all training trials, and the orange line depicts the function generated by the network on a particular test trial.

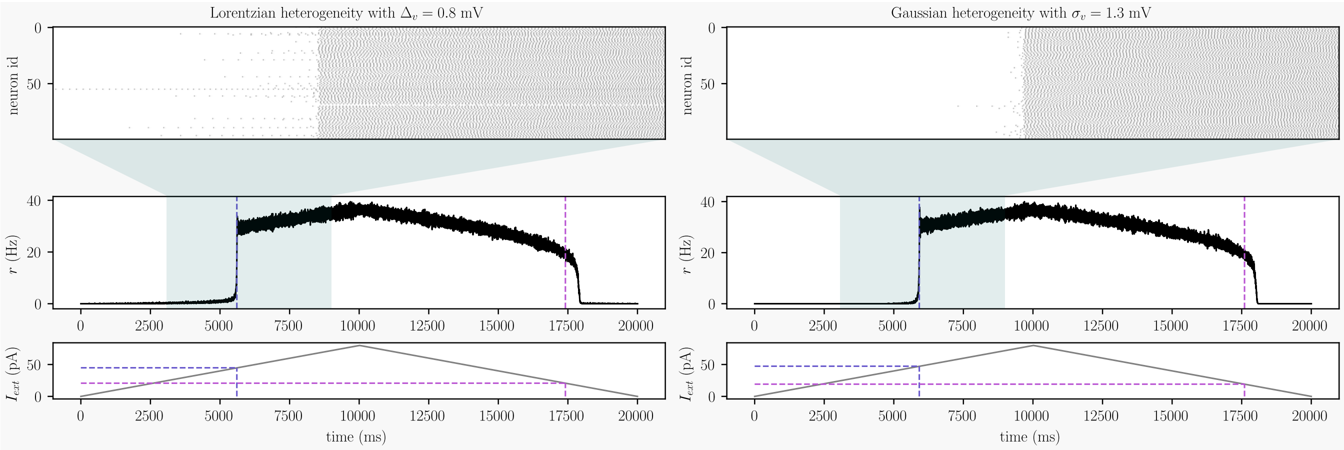

Fig. S10. Fold bifurcation detection in a population of regular spiking neurons. The left and right column depict results for Lorentzian and Gaussian distributions of spike thresholds, respectively. **Top row:** Spiking activity of 100 exemplary neurons around a fold bifurcation. **Middle row:** Average firing rate dynamics in the network in response to an input ramp. Horizontal dashed lines represent the approximate locations of fold bifurcation points. The shaded time interval depicts the time interval that corresponds to the spike raster plot. **Bottom row:** Depiction of the input ramp over time. Dashed lines depict the time point and input value for which fold bifurcations were detected.

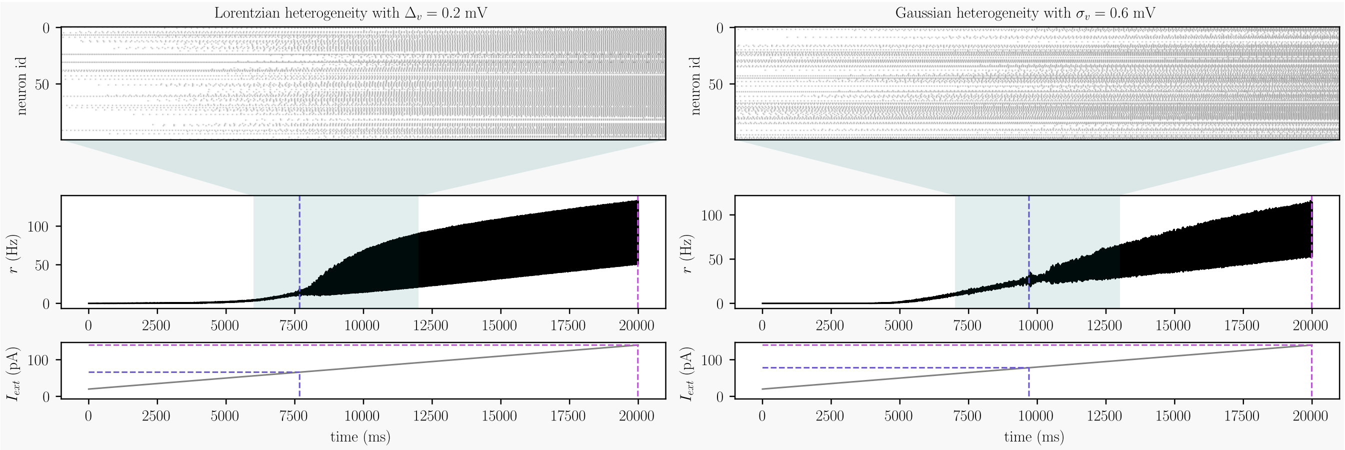

Fig. S11. Hopf bifurcation detection in a population of fast spiking neurons. The left and right column depict results for Lorentzian and Gaussian distributions of spike thresholds, respectively. **Top row:** Spiking activity of 100 exemplary neurons around a Hopf bifurcation. **Middle row:** Average firing rate dynamics in the network in response to an input ramp. Horizontal dashed lines represent the approximate locations of Hopf bifurcation points. The shaded time interval depicts the time interval that corresponds to the spike raster plot. **Bottom row:** Depiction of the input ramp over time. Dashed lines depict the time point and input value for which Hopf bifurcations were detected.

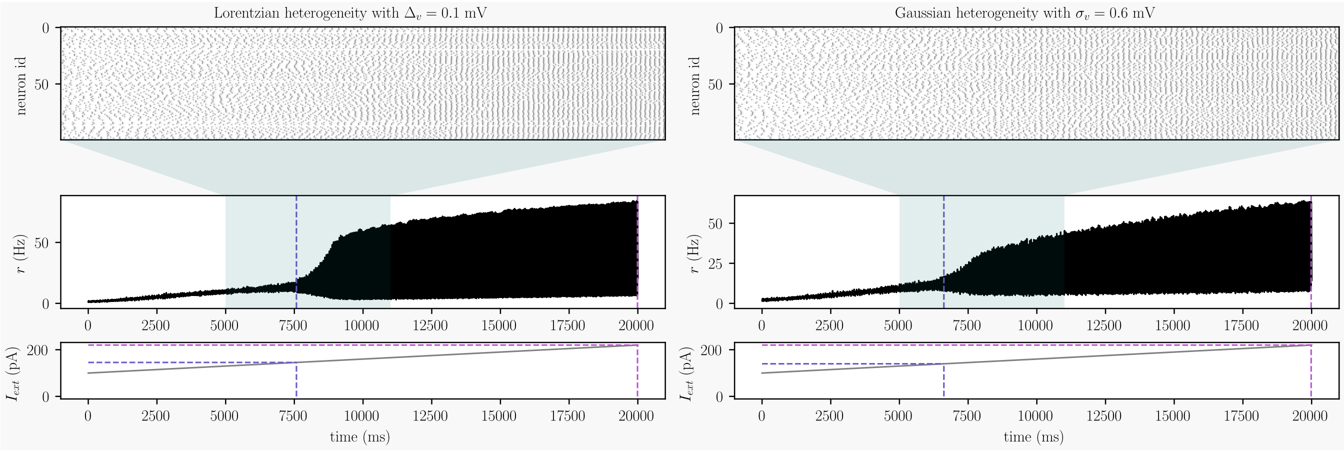

Fig. S12. Hopf bifurcation detection in a population of low-threshold spiking neurons. The left and right column depict results for Lorentzian and Gaussian distributions of spike thresholds, respectively. **Top row:** Spiking activity of 100 exemplary neurons around a Hopf bifurcation. **Middle row:** Average firing rate dynamics in the network in response to an input ramp. Horizontal dashed lines represent the approximate locations of Hopf bifurcation points. The shaded time interval depicts the time interval that corresponds to the spike raster plot. **Bottom row:** Depiction of the input ramp over time. Dashed lines depict the time point and input value for which Hopf bifurcations were detected.

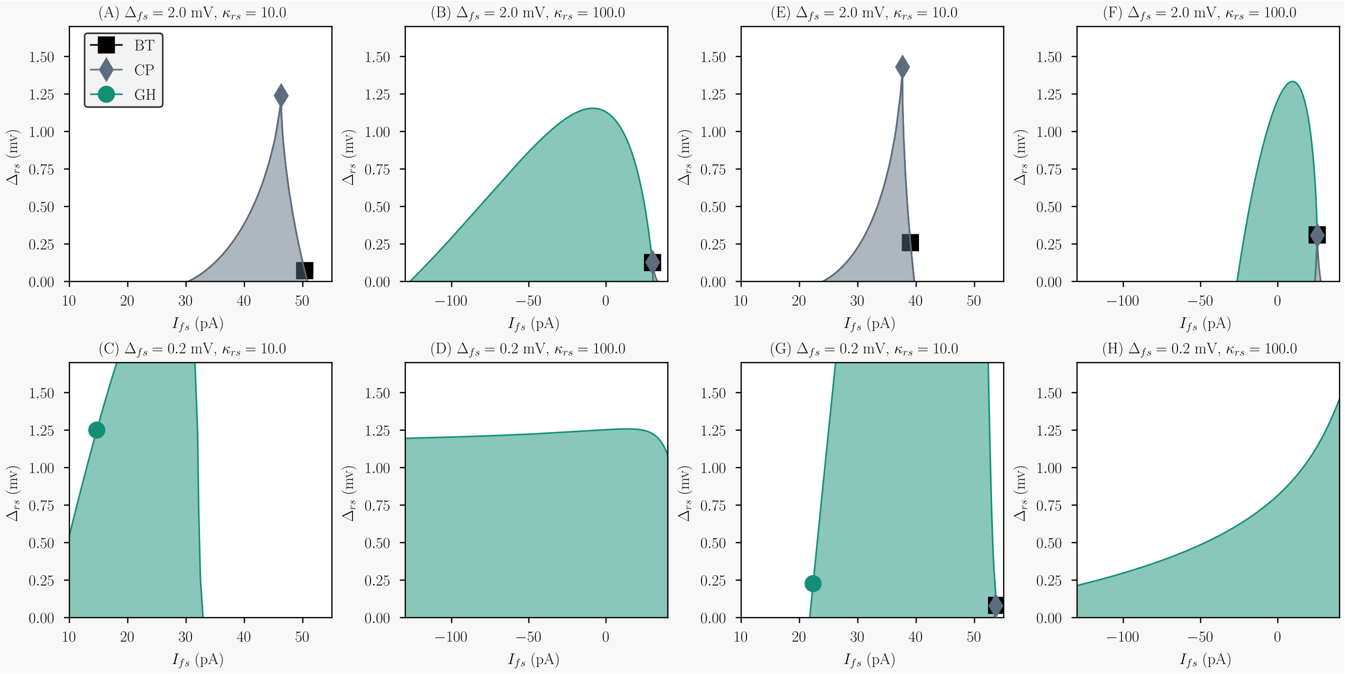

Fig. S13. Effects of inhibitory interneuron heterogeneity in a two-population model for different coupling patterns. **A-D** Bifurcation structure of a two-population model of coupled regular spiking and fast spiking neurons with the following coupling parameters: $k_{rr} = 14$, $k_{rf} = 14$, $k_{fr} = 7$, $k_{ff} = 7$. **E-H** Bifurcation structure of a two-population model of coupled regular spiking and fast spiking neurons with the following coupling parameters: $k_{rr} = 16$, $k_{rf} = 16$, $k_{fr} = 4$, $k_{ff} = 0$. Regions shaded in grey depict bistable regimes and are enclosed by fold bifurcation curves (grey lines). Regions shaded in green depict oscillatory regimes and are enclosed by Hopf bifurcation curves (green lines). Black squares depict Bogdanov-Takens bifurcations, grey diamonds depict cusp bifurcations, and green circles depict generalized Hopf bifurcations. Bifurcation diagrams in the top row were obtained for heterogeneous fast spiking neurons ($\Delta_{fs} = 2$ mV), whereas bifurcation diagrams in the bottom row were obtained for homogeneous fast spiking neurons ($\Delta_{fs} = 0.2$ mV). For each condition (defined by the fast spiking neuron heterogeneity and the set of coupling parameters), we provide bifurcation diagrams for regular spiking neurons with low spike frequency adaptation ($\kappa_{rs} = 10$ pA) and high spike frequency adaptation ($\kappa_{rs} = 100$ pA).

References

1. E Izhikevich, Simple model of spiking neurons. *IEEE Transactions on Neural Networks* **14**, 1569–1572 (2003) Conference Name: IEEE Transactions on Neural Networks.
2. E Montbrió, D Pazó, A Roxin, Macroscopic Description for Networks of Spiking Neurons. *Phys. Rev. X* **5**, 021028 (2015).
3. L Chen, SA Campbell, Exact mean-field models for spiking neural networks with adaptation. *J. Comput. Neurosci.* **50**, 445–469 (2022).
4. R Gast, SA Solla, A Kennedy, Macroscopic dynamics of neural networks with heterogeneous spiking thresholds. *Phys. Rev. E* **107**, 024306 (2023).
5. C Buetfering, K Allen, H Monyer, Parvalbumin interneurons provide grid cell–driven recurrent inhibition in the medial entorhinal cortex. *Nat. Neurosci.* **17**, 710–718 (2014) Number: 5 Publisher: Nature Publishing Group.
6. R Gast, SA Solla, A Kennedy, Effects of Neural Heterogeneity on Spiking Neural Network Dynamics (2022) Number: arXiv:2206.08813 arXiv:2206.08813 [nlin, q-bio].
7. Y Wang, et al., Anatomical, physiological and molecular properties of Martinotti cells in the somatosensory cortex of the juvenile rat. *The J. Physiol.* **561**, 65–90 (2004).
8. D Lau, et al., Impaired Fast-Spiking, Suppressed Cortical Inhibition, and Increased Susceptibility to Seizures in Mice Lacking Kv3.2 K+ Channel Proteins. *J. Neurosci.* **20**, 9071–9085 (2000).
